# Integrated Analysis Identifies an Immune-Based Prognostic Signature for the Mesenchymal Identity in Gastric Cancer

**DOI:** 10.1155/2020/9780981

**Published:** 2020-04-09

**Authors:** Dongzhu Peng, Bin Gu, Liming Ruan, Xingguo Zhang, Peng Shu

**Affiliations:** ^1^Department of Gastroenterology, Beilun People's Hospital, Ningbo, China; ^2^Department of Gastrointestinal Hernia Surgery, Cangzhou People's Hospital, Cangzhou, China; ^3^Molecular Laboratory, Beilun People's Hospital, Ningbo, China

## Abstract

**Background:**

Gastric cancer (GC) has been divided into four molecular subtypes, of which the mesenchymal subtype has the poorest survival. Our goal is to develop a prognostic signature by integrating the immune system and molecular modalities involved in the mesenchymal subtype.

**Methods:**

The gene expression profiles collected from 6 public datasets were applied to this study, including 1,221 samples totally. Network analysis was applied to integrate the mesenchymal modalities and immune signature to establish an immune-based prognostic signature for GC (IPSGC).

**Results:**

We identified six immune genes as key factors of the mesenchymal subtype and established the IPSGC. The IPSGC can significantly divide patients into high- and low-risk groups in terms of overall survival (OS) and relapse-free survival (RFS) in discovery (OS: *P* < 0.001) and 5 independent validation sets (OS range: *P* = 0.05 to *P* < 0.001; RFS range: *P* = 0.03 to *P* < 0.001). Further, in multivariate analysis, the IPSGC remained an independent predictor of prognosis and performed better efficiency compared to clinical characteristics. Moreover, macrophage M2 was significantly enriched in the high-risk group, while plasma cells were enriched in the low-risk group.

**Conclusions:**

We propose an immune-based signature identified by network analysis, which is a promising prognostic biomarker and help for the selection of GC patients who might benefit from more rigorous therapies. Further prospective studies are warranted to test and validate its efficiency for clinical application.

## 1. Introduction

Gastric cancer (GC) is ranked as the third cause of cancer-related death; each year, there is about one million newly diagnosed GC [1, 2]. In the early stage of GC, surgery can prolong the survival of patients [3]. However, more than half of the patients with advanced-stage GC have local recurrence or distant metastasis which eventually leads to poor prognosis (5-year survival rate is about 5-10%) [3, 4]. Therefore, researchers and clinicians need to focus on targeted prognostic and treatment strategies and accurately identify and personalize treatments to extend GC patient survival.

Gene expression-based biomarkers in tumor tissue are reliably associated with cancer prognosis [5, 6]. Large-scale public cohorts with tumor gene expression data provide a broader opportunity to search for reliable prognostic markers for gastric cancer. Several studies have developed markers based on gene expression for GC prognosis prediction [7–10]. However, due to the heterogeneity of GC, most of the markers have low prognostic efficacy and cannot be directly used in clinical practice. Recently, four gastric cancer subtypes with different molecular and clinical characteristics were found [11], among which the mesenchymal subtype had the poorest prognosis. Thus, the intrinsic modalities of the more malignant mesenchymal subtype could potentially be used for risk assessment in GC patients and for development of more precise and targeted treatment strategies.

There is growing evidence that the immune system plays an important role in the development and progression of cancer [12, 13]. Many previous studies have shown that immunotherapy targeting immune checkpoint is strongly pursued [14, 15]. In addition, previous studies have tentatively shown that the immune system has a prognostic value in gastric cancer [16, 17]. Therefore, it is possible to develop prognostic markers based on immune genes for clinical application in gastric cancer.

In this study, we applied network analysis to integrate mesenchymal modalities and immune signature genes from the ImmPort database underlying the mesenchymal subtype. The master regulator analysis showed that six immune genes were the key factors of the mesenchymal subtype. We pooled and analyzed six public cohorts containing 1,221 GC patients to develop and validate an immune gene-based prognostic signature for GC (IPSGC) using these six immune genes. Although immune prognostic markers for gastric cancer have been reported [18], no research has been done for risk stratification by integrating the characteristics of the mesenchymal subtype. The robustness and reliability of our model were proved by sufficient verification in 5 independent datasets. In addition, we conducted a comprehensive analysis to investigate the intrinsic biological and clinical relevance of IPSGC. Our signature combines the molecular modalities involved in the mesenchymal subtype and would be used to screen GC patients who may benefit from more rigorous treatment.

## 2. Materials and Methods

### 2.1. Ethical Approval

The researchers were authorized to conduct the study by the Ethics Committee of the Beilun People's Hospital, Ningbo, China. All procedures were implemented in accordance with the Declaration of Helsinki and relevant policies in China.

### 2.2. Patient Series

**Figure 1:**
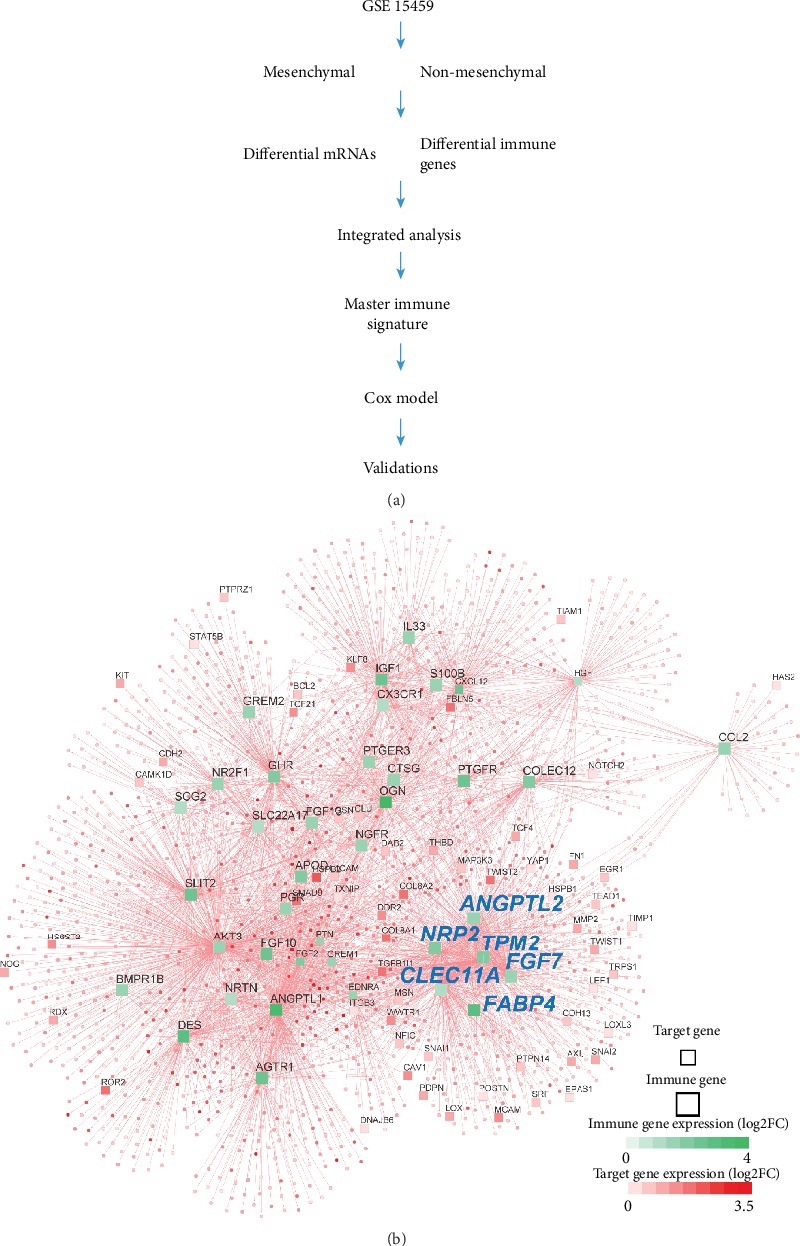
Network inference identifies six immune signature genes as key regulators of the mesenchymal subtype in GC. (a) Study design. (b) The integrated network displays the relationships between the six immune signature genes and the regulated EMT signature genes.

We retrospectively collected and comprehensively analyzed the gene expression profiles (GEPs) from 6 independent datasets, containing 1,221 cases. The complete lists of all GEPs are shown in Table S1. These datasets involved patients from GSE15459 (*n* = 192), GSE13861 (*n* = 65), GSE84437 (*n* = 433), GSE62254 (*n* = 300), GSE26901 (*n* = 97), and GSE29272 (*n* = 134). The expression data of all cohorts together with the corresponding clinical parameters were downloaded from Gene Expression Omnibus (GEO). The molecular subtyping information for GSE15459 and GSE62254 was retrieved from Cristescu et al.'s study [11]. The detailed clinical characteristics of the 6 datasets were described in Table 1. The design and workflow of this study are illustrated in Figure 1(a).

### 2.3. Expression Data Preprocessing

GEPs were downloaded from GEO by “GEOquery” (R package, version 1.0.7) [19] and normalized with the robust multiarray analysis (RMA). For each cohort, the GEPs were collapsed from probe IDs to gene symbols; if multiple probe IDs correspond to the same gene symbol, the one with the highest mean value was kept as the representative of the corresponding gene [20].

### 2.4. Integrated Network Analysis

Immune genes (IRGs) were downloaded from the ImmPort database [21]. IRGs measured by all cohorts were kept. Network analysis was applied to integrate mesenchymal modalities and immune genes underlying the mesenchymal subtype. Together, we used the GSE15459 dataset as the training cohort. 46 immune genes (|log2FC∣ > 1.5, FDR < 0.05) and 1,865 target genes (log2FC > 0.5, FDR < 0.05) were differentially expressed by comparing the mesenchymal subtype with the other three subtypes (MSI, TP53-, and TP53+). Integrated network analysis was performed by “RTN” (R package, version 2.10.0) [22]. Master regulator analysis (MRA) was done to examine significantly overrepresented epithelial-mesenchymal transition (EMT) genes [23] within each immune gene's regulon. Six immune genes of top significance were kept as the key factors of the mesenchymal subtype.

### 2.5. Development of the Immune-Based Prognostic Signature for GC (IPSGC)

Six immune genes are differentially upexpressed in the poorest survival subtype and are the master regulatory factors of the mesenchymal subtype-specific genes (including EMT genes). The Cox proportional hazards model was applied to test their association with overall survival. Based on these six immune genes, we develop a Cox model named the immune-based prognostic signature for GC (IPSGC) as follows: risk score = (0.3686 × CLEC11A) + (0.0545 × NRP2) + (0.3192 × TPM2) + (−0.1722 × ANGPTL2) + (−0.1892 × FGF7) + (−0.0768 × FABP4).

### 2.6. Validation of the IPSGC

The IPSGC score was further evaluated in the 5 independent validation cohorts in terms of OS and RFS by the log-rank test, respectively. The IPSGC then was evaluated with other clinical parameters in the uni- and multivariate Cox analyses. In the multivariable Cox regression, tumor stages, histology, and gender were included as covariates.

### 2.7. Profiling of Immune Cell Infiltration

To analyze the immunobiological characteristics of the high- and low-risk groups, we used CIBERSORT [24], to characterize immune cells' abundance of tumor tissue GEPs. Based on a set of reference immune cell GEPs, CIBERSORT used support vector regression [24] to deconvolute tumor tissue gene expression profile with each type of immune cell enrichment. More specifically, standardized gene expression profiles were submitted to the CIBERSORT Web portal (http://cibersort.stanford.edu/) with 1,000 permutations. For each sample, CIBERSORT quantified the relative proportions of 22 infiltrated immune cell types.

### 2.8. Gene Ontology (GO) Analysis and Gene Set Enrichment Analysis (GSEA)

GO analysis was conducted for the significantly upregulated genes in the high-risk group using g:Profiler [25]. GSEA [26] was conducted using “fgsea” (Bioconductor package, version 1.12.0) with 1,000 permutations. Gene sets were retrieved from the Molecular Signature Database (MSigDB hallmark and KEGG, version 7) [26]. A *P* value below 0.05 was used to choose significant gene sets.

### 2.9. Statistical Analysis

Continuous variables were compared using the Wilcoxon signed-rank test or Kruskal-Wallis rank-sum test. Kaplan-Meier analysis was performed using the log-rank test using “survival” (R package, version 2.41.3). Uni- and multivariable analyses were conducted by the Cox proportional hazards model. For all tests, a *P* value below 0.05 was used to choose significant gene sets. Statistical significance is presented as follows: ^∗^*P* < 0.05, ^∗∗^*P* < 0.01, and ^∗∗∗^*P* < 0.001. All the statistical tests were conducted using R (version 3.6.1).

## 3. Results

### 3.1. Integrative Analysis Reveals Seven Immune Genes as Master Regulators for the Mesenchymal Subtype of GC

GC is a molecularly heterogeneous disease. In recent studies [11], four molecular subtypes have been identified, among which the mesenchymal subtype has the worst prognosis (Figure S1(A, B)). To investigate the immune system role underlying the mesenchymal subtype, a total of 1,221 patients with GC from 6 independent public cohorts were included (Table 1). We applied network analysis to integrate mesenchymal modalities and immune genes in the GSE15459 cohort (Figure 1(a)). We investigated the differences between the mesenchymal subtype and the other three subtypes, as the mesenchymal subtype has shown the worst prognostic outcome. The networks consist of 46 immune genes (∣log2FC∣ > 1.5, FDR < 0.05) and 1,865 target genes (log2FC > 0.5, FDR < 0.05) by comparing the mesenchymal subtype with the other three subtypes (Figure 1(b)). Master regulator analysis (MRA) identified six immune genes (CLEC11A, NRP2, TPM2, ANGPTL2, FGF7, and FABP4) as the key factors of the mesenchymal subtype (Table S2). These six immune genes are significantly upexpressed in the mesenchymal subtype in the training cohort and one validation dataset containing molecular subtyping information (Figure S2). FN1 [27], SNAI1 [28], TGFB1 [29], and CDH1 [30], which are epithelial-mesenchymal transition (EMT) signature [23] genes, are significantly correlated with the six immune genes, showing that the mesenchymal property is indeed governed by these six immune genes (Figure S3). Results from the univariable Cox proportional analysis demonstrated strong prognostic values of the six immune genes for OS (Figure S4). Therefore, these six immune genes are key factors of the mesenchymal modalities and can be potentially applied for risk assessment of GC patients.

### 3.2. Development of the Immune-Based Prognostic Signature for GC (IPSGC)

**Figure 2:**
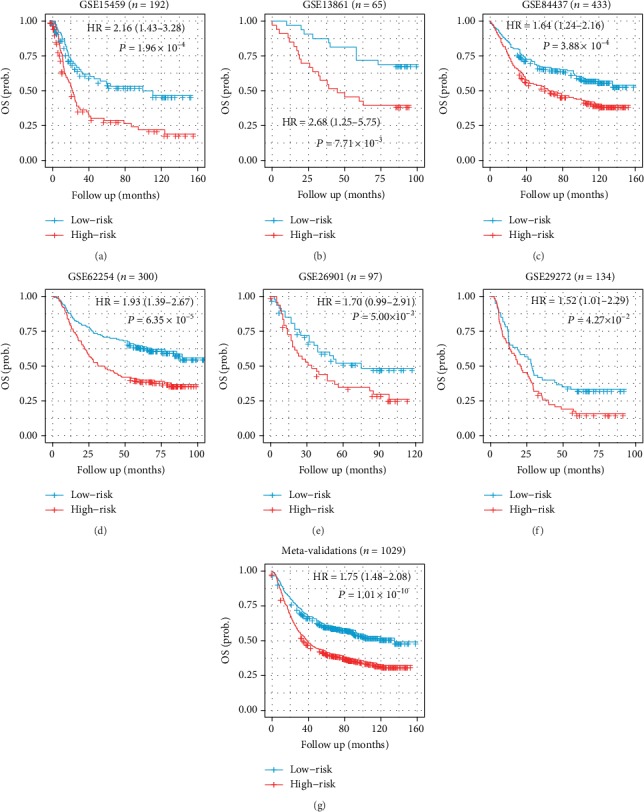
Prognostic value of the IPSGC. Kaplan-Meier plots showing differences in overall survival among different risk groups stratified by the median risk score within (a) GSE15459, (b) GSE13861, (c) GSE84437, (d) GSE62254, (e) GSE26901, (f) GSE29272, and (g) the combination of all validation cohorts. *P* values are calculated by the log-rank tests.

Using the GSE15459 cohort as the training set, we defined the IPSGC using Lasso Cox proportional hazards regression of these seven immune genes: risk score = (0.3686 × CLEC11A) + (0.0545 × NRP2) + (0.3192 × TPM2) + (−0.1722 × ANGPTL2) + (−0.1892 × FGF7) + (−0.0768 × FABP4). Risk scores were calculated in all the training and validation cohorts (Table S3). The risk score plots clearly demonstrate the difference between alive and dead patients (Figure S5(A, B)). The median risk value was set as the cutoff to separate patients into high- and low-risk groups across all datasets. In the training set, the high-risk group displayed a worse OS (HR: 2.16, 95% CI: 1.43− 3.28; *P* = 1.96 × 10^−4^) (Figure 2(a) and Table S3).

### 3.3. Validation of the IPSGC

**Figure 3:**
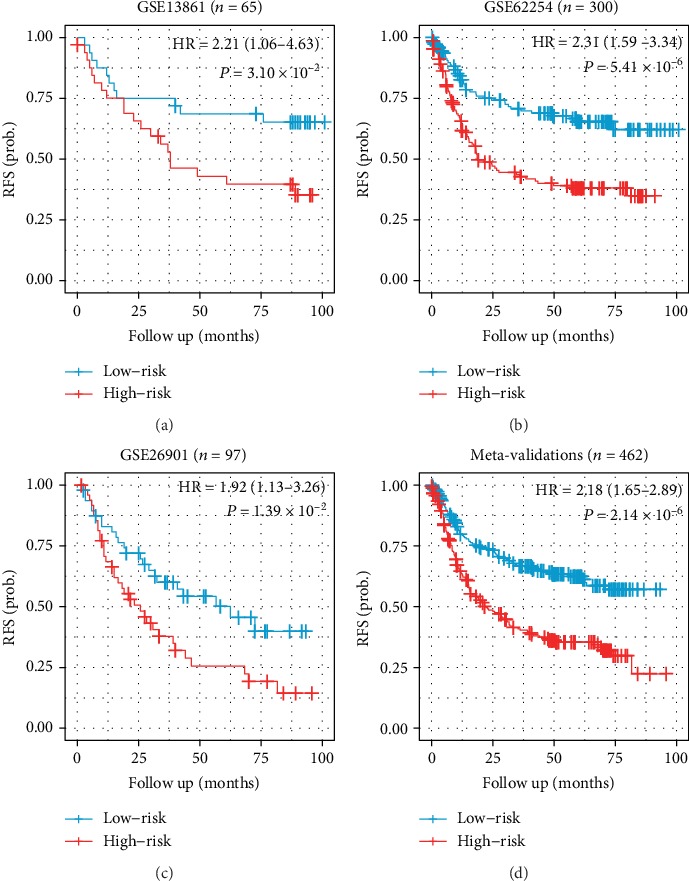
Kaplan-Meier plots showing differences in relapse-free survival among different risk groups within (a) GSE13861, (b) GSE62254, (c) GSE26901, and (d) the combination of the three cohorts. *P* values are based on log-rank tests.

To verify the prognostic power of the IPSGC, we calculated the survival difference within the two risk groups in 5 validation cohorts. As expected, the IPSGC significantly stratified patients into high- and low-risk groups in terms of OS (HR range: 1.70 [95% CI: 0.99–2.91; *P* = 5.00 × 10^−2^] to 1.93 [95% CI: 1.39–2.67; *P* = 6.35 × 10^−5^]) (Figures 2(b)–2(f) and Table S3) and RFS (HR range: 2.21 [95% CI: 1.06–4.63; *P* = 3.10 × 10^−2^] to 2.31 [95% CI: 1.59–3.34; *P* = 5.41 × 10^−6^]) (Figures 3(a)–3(c)) and Table S3) in the 5 validation cohorts. In the meta-analysis for all datasets, the prognostic effects of the IPSGC are more obvious in terms of OS (HR: 1.75, 95% CI: 1.48− 2.08; *P* = 1.01 × 10^−10^) (Figure 2(g)) and RFS (HR: 2.18, 95% CI: 1.65− 2.89; *P* = 2.14 × 10^−6^) (Figure 3(d)). Furthermore, it remains an independent predictor of prognosis in the uni- and multivariate Cox models, after adjusting for stage, histology, and gender (Table 2).

### 3.4. In Silico Functional Assessment of the IPSGC

**Figure 4:**
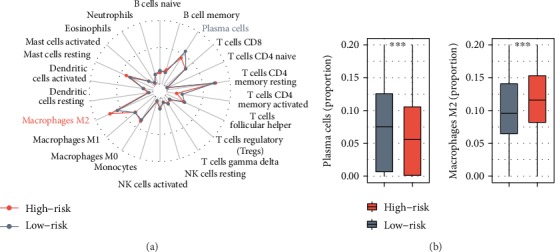
Immune infiltration status between the two immune-risk groups. (a) Immune infiltration status for different immune-risk groups. (b) The proportion level of plasma and monocytes for different immune-risk groups. For every immune cell subset, the Wilcoxon test *P* value comparing the high- vs. low-immune-risk groups are shown. ^∗∗^*P* < 0.01 and ^∗∗∗^*P* < 0.001.

**Figure 5:**
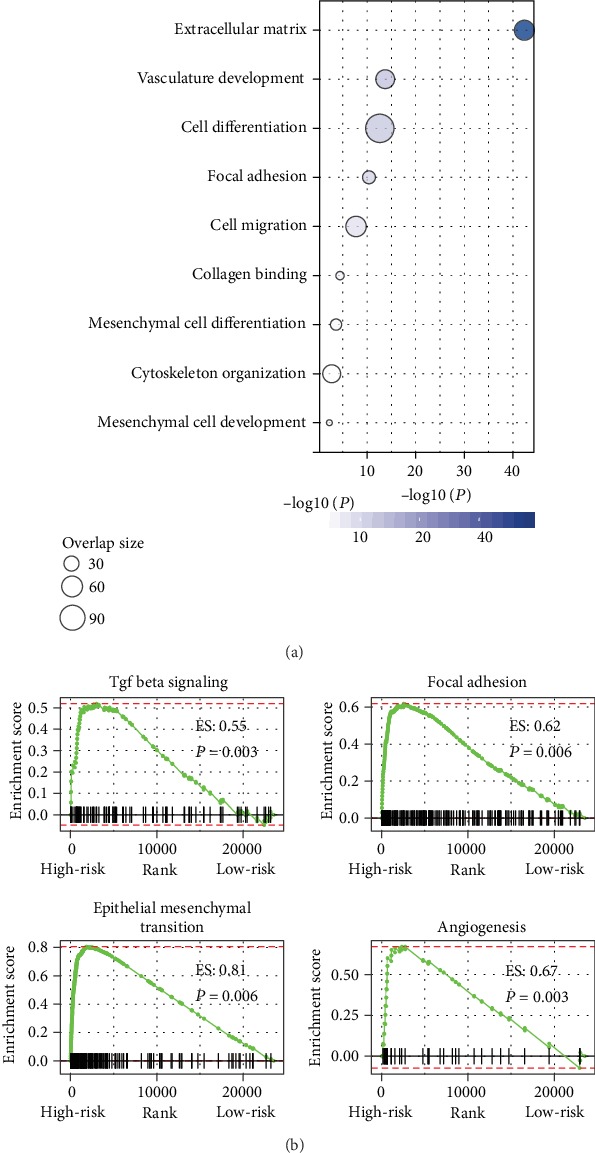
Pathway alternation between the two immune-risk groups. (a) Gene ontology (GO) of the upexpressed genes between the high- and low-immune-risk groups. (b) Gene Set Enrichment Analysis (GSEA) between the high- and low-immune-risk groups.

To gain insight into the biological differences between risk groups, we performed immune cell infiltration, GO, and GSEA analyses. Immune cell types, such as macrophages M2, T cell CD4 memory resting, and T cell CD8, were enriched in training and validation cohorts (Figure S6). We observed a significantly higher proportion of macrophage M2 in the high-risk group and a significantly higher enrichment of plasma cells in the low-risk group (Figures 4(a) and 4(b)). Furthermore, these two risk groups' specific immune cell infiltration was also validated in validation cohorts (Figure S7). GO analysis showed that the differentially expressed genes between risk groups were mostly involved in immune responses and tumor metastasis (Figure 5(a)). Enrichment analysis between high- and low-risk groups identified that many mesenchymal phenotype-related pathways, including the TGF-beta signaling, epithelial-mesenchymal transition, angiogenesis, and focal adhesion, were positively enriched in the high-risk group (*P* < 0.01) (Figure 5(b) and Table S4). We observed that the risk score levels were significantly increased with tumor stages, of which stage IV patients have the highest risk score (Figure S8(A)). Moreover, the risk scores were significantly higher in diffuse GC than in other GC histologies (Figure S8(B)).

## 4. Discussion

Gastric cancer (GC) is the third leading cause of cancer death with pathological and molecular heterogeneity characteristics [1, 2, 11]. In the past, clinicopathological indicators have been used for risk stratification of GC. However, some patients with advanced GC remained stable for several years after surgery, while some patients with early GC progressed rapidly [31]. At present, various multigene prognostic markers have been developed [7–10], but their prediction efficiencies were still uncertain. Therefore, a new signature that can accurately recognize patients with poor GC prognosis is urgently needed to give more rigorous treatments.

The GC transcriptome was unsupervised classified into four molecular subtypes with different molecular and clinical characteristics [11]. Prognostic signature screened based on molecular portraits specific to the worst prognosis subtype may be used for risk stratification of GC patients [32, 33]. Recent studies have shown that the tumor microenvironment plays an important role in the occurrence and development of tumors [34]. The occasion, growth, and forecast of GC are closely related to the crosstalk between different immune cells and GC cells [35]. In this study, we established an immune gene-based prognostic signature for GC (IPSGC) by integrating mesenchymal modalities and the immune system underlying the mesenchymal subtype and validated it in 5 independent validation cohorts which covered most common microarray platforms. The large sample size provided sufficient validation for the IPSGC and makes it more robust. To our knowledge, no research has been done for risk stratification by integrating the immune system and the characteristics of the mesenchymal subtype in GC. The IPSGC was constructed by six immune genes as the key factors of the mesenchymal subtype and could stratify patients into different risk groups. Within these six immune genes, CLEC11A was the driver gene in multiple myeloma (MM) [36]. NRP2 could promote gastric adenocarcinoma lymphatic invasion with VEGF-C stimulation [37]. High expression of ANGPTL2 is associated with tumor malignancy, early recurrence, and poor prognosis in GC patients [38]. FGF7 promotes gastric cancer invasion and migration [39]. Elevated expression of FABP4 correlates with poor prognosis in non-small-cell lung cancer (NSCLC) [40]. The defined high-risk group showed a worse OS and RFS than the low-risk group. The IPSGC remained an independent prognostic predictor in multivariate Cox proportional hazards analysis after adjusting for other clinical factors. Most genes within the differentially expressed genes between risk groups were mostly involved in immune responses and tumor metastasis. Previous studies have described that the infiltration of plasma cells contributes to prolonged survival in GC [41], while macrophage M2 indicates a poor prognosis in GC [42]. We observed a significantly higher proportion of macrophage M2 in the high-risk group and a significantly higher enrichment of plasma cells in the low-risk group. Moreover, some mesenchymal phenotype-related pathways, such as EMT, angiogenesis, and focal adhesion, were positively enriched in the high-risk group. Our findings inferred the important role of IPSGC in tumor invasion and therefore could sever as a robust prognostic signature in GC.

This study still has some limitations. First of all, the prognostic signature was screened from gene expression profiles generated from microarray platforms, which are expensive, are difficult to operate, and involve professional bioinformatics expertise, so it is difficult to be popularized in daily clinical application. Second, the training and validation datasets were all from retrospective studies in the study, including fresh frozen samples. Therefore, the efficiency and stability of FFPE samples are still in doubt. In the following improvement process, more datasets containing more clinical characteristics need to be included for more extensive screening and validation.

## 5. Conclusion

Taken together, our network analysis established an immune gene-based signature, which could effectively predict GC patients' survival. Our study is the first attempt to integrate tumor heterogeneity and the immune system to develop the prognostic signature for GC.

## Figures and Tables

**Table 1:** Patients' characteristics in public datasets.

	Training cohort	Validation cohorts				
	GSE15459 (*n* = 192)	GSE13861 (*n* = 65)	GSE84437 (*n* = 433)	GSE62254 (*n* = 300)	GSE26901 (*n* = 97)	GSE29272 (*n* = 134)
Age (years)	64 (23-92)	61 (32-83)	60 (27-86)	62 (24-86)	56 (28-74)	56 (23-73)
Gender						
Female	67 (35%)	19 (29%)	137 (32%)	101 (34%)	37 (38%)	31 (23%)
Male	125 (65%)	46 (71%)	296 (68%)	199 (66%)	60 (62%)	103 (77%)
Histology						
Diffuse	75 (39%)	30 (46%)		135 (45%)	10 (10%)	
Indeterminate				2 (1%)		
Intestinal	99 (52%)	19 (29%)		146 (49%)	71 (73%)	
Mixed	18 (9%)	12 (18%)		17 (6%)	5 (5%)	
Unknown		4 (6%)			11 (11%)	
Site						
Antrum		26 (40%)		152 (51%)	51 (53%)	
Body		31 (48%)		110 (37%)	30 (31%)	
Cardia		4 (6%)		32 (11%)		
Entire		1 (2%)		3 (1%)	4 (4%)	
Fundus		1 (2%)			12 (12%)	
Unknown		2 (3%)				
Stage						
I	31 (16%)	12 (18%)		31 (10%)	33 (34%)	5 (4%)
II	29 (15%)	12 (18%)		97 (32%)	17 (18%)	5 (4%)
III	72 (38%)	25 (38%)		95 (32%)	32 (33%)	115 (86%)
IV	60 (31%)	16 (25%)		77 (26%)	15 (15%)	9 (7%)

**Table 2:** Univariate and multivariate analyses of immune signature and clinicopathological factors.

	Metavalidation cohorts			
	Univariate		Multivariate	
	HR (95% CI)	*P*	HR (95% CI)	*P*
Gender (male vs. female)	1.09 (0.90-1.33)	0.36	1.02 (0.75-1.39)	0.9
Stage (III & IV vs. I & II)	3.62 (2.64-4.96)	1.24*E*-15	3.32 (2.32-4.75)	5.2*E*-11
Histology (diffuse & others)	1.38 (1.03-1.84)	0.03	1.23 (0.91-1.66)	0.18
Immune signature (high vs. low risk)	1.76 (1.46-2.11)	1.34*E*-09	1.93 (1.42-2.62)	2.5*E*-05

## Data Availability

The data used to support the findings of this study are available from the corresponding author upon request.

## Supplementary Materials

Supplementary 1 Fig S1: Kaplan-Meier plots among different molecular subtypes. (A) GSE15459 and (B) GSE62254.

Supplementary 2 Fig S2: the expression level of the six immune signature genes across the four molecular subtypes in different cohorts.

Supplementary 3 Fig S3: association between the six immune signature genes and EMT signature genes in the different molecular subtypes. (A) FN1, (B) TGFB1, and (C) SNAI1, (D) CDH1.

Supplementary 4 Fig S4: prognostic associations of the six immune signature genes. *P* values are based on the uni-Cox analysis. ^∗^*P* < 0.05, ^∗∗^*P* < 0.01, and ^∗∗∗^*P* < 0.001.

Supplementary 5 Fig S5: risk-score plots for the training cohort GSE15459 (A) and one validation cohort GSE62254 (B).

Supplementary 6 Fig S6: summary of inferred immune cell subset proportions across different datasets.

Supplementary 7 Fig S7: the proportion level of plasma (A) and macrophage M2 (B) for different immune-risk groups in different cohorts.

Supplementary 8 Fig S8: the clinical relevance of the IPSGC. (A) The risk score across tumor stages in different datasets (left: GSE13861; middle: GSE15459; and right: GSE62254). (B) The risk score for histology types in GSE15459.

Supplementary 9 Table S1: details about the public datasets used in this study.

Supplementary 10 Table S2: master regulated analysis results.

Supplementary 11 Table S3: patients risk stratification.

Supplementary 12 Table S4: GSEA results for the comparison of high- vs. low-risk groups.
