# Supplementary material for: Integrated Analysis Identifies an Immune-Based Prognostic Signature for the Mesenchymal Identity in Gastric Cancer
Source: Biomed Res Int. 2020 Apr 9;2020:9780981. doi: 10.1155/2020/9780981 (PMC7171688; doi:10.1155/2020/9780981)
Supplement: Supplementary 9 — Table S1: details about the public datasets used in this study. [file 9780981.f9.docx]

Table S1. Details about the data sets used in this study

| **Patient data** | **Samples** | **Platform** | **Reference** |
| --- | --- | --- | --- |
| GSE15459 | 192 | Affymetrix Human Genome U133 Plus 2.0 Array | PMID: 19798449 |
| GSE13861 | 65 | Illumina HumanWG-6 v3.0 expression beadchip | PMID: 21447720 |
| GSE84437 | 433 | Illumina HumanHT-12 V3.0 expression beadchip |  |
| GSE62254 | 300 | Affymetrix Human Genome U133 Plus 2.0 Array | PMID: 25894828 |
| GSE26901 | 97 | Illumina HumanHT-12 V3.0 expression beadchip | PMID: 29725014 |
| GSE29272 | 134 | Affymetrix Human Genome U133A Array | PMID: 23717493 |
