# Supplementary material for: Integrated Analysis Identifies an Immune-Based Prognostic Signature for the Mesenchymal Identity in Gastric Cancer
Source: Biomed Res Int. 2020 Apr 9;2020:9780981. doi: 10.1155/2020/9780981 (PMC7171688; doi:10.1155/2020/9780981)
Supplement: Supplementary 10 — Table S2: master regulated analysis results. [file 9780981.f10.docx]

Table S2. Master regulated analysis results

| **Regulator** | **Regulon Size** | ***P*-value** |
| --- | --- | --- |
| ANGPTL2 | 161 | 0.011 |
| CLEC11A | 147 | 0.0052 |
| FGF7 | 88 | 0.012 |
| FABP4 | 22 | 0.042 |
| NRP2 | 123 | 0.03 |
| TPM2 | 131 | 0.043 |
| TNC | 15 | 0.1 |
| AGTR1 | 117 | 0.93 |
| AKT3 | 397 | 0.7 |
| ANGPTL1 | 220 | 0.7 |
| APOD | 18 | 1 |
| BMPR1B | 22 | 1 |
| CCL2 | 54 | 0.59 |
| COLEC12 | 92 | 0.43 |
| CTSG | 32 | 1 |
| CX3CR1 | 74 | 0.76 |
| CXCL12 | 132 | 0.72 |
| DES | 122 | 1 |
| EDNRA | 66 | 0.43 |
| ELN | 57 | 0.34 |
| FGF10 | 29 | 0.28 |
| FGF13 | 49 | 0.84 |
| FGF2 | 104 | 0.74 |
| GHR | 177 | 0.96 |
| GREM1 | 32 | 0.69 |
| GREM2 | 29 | 0.28 |
| HGF | 102 | 0.98 |
| IGF1 | 132 | 0.87 |
| IL33 | 40 | 0.77 |
| NGFR | 48 | 1 |
| NR2F1 | 76 | 0.77 |
| NRTN | 98 | 0.98 |
| OGN | 152 | 0.81 |
| PDGFRL | 65 | 0.91 |
| PGR | 60 | 0.89 |
| PTGER3 | 31 | 1 |
| PTGFR | 66 | 0.43 |
| PTN | 51 | 0.56 |
| S100B | 38 | 0.76 |
| SCG2 | 47 | 0.83 |
| SLC22A17 | 72 | 0.74 |
| SLIT2 | 153 | 1 |
| TAC1 | 31 | 1 |
