# Supplementary material for: Integrated Analysis Identifies an Immune-Based Prognostic Signature for the Mesenchymal Identity in Gastric Cancer
Source: Biomed Res Int. 2020 Apr 9;2020:9780981. doi: 10.1155/2020/9780981 (PMC7171688; doi:10.1155/2020/9780981)
Supplement: Supplementary 11 — Table S3: patients risk stratification. [file 9780981.f11.docx]

Table S3. Patients risk stratification

| **Cohort** | **Samples** | **Age** | **Histological** | **Stage** | **Subtype** | **Risk group** |
| --- | --- | --- | --- | --- | --- | --- |
| GSE15459 | GSM387789 | 76 | Mixed | 4 | EMT | high-risk |
| GSE15459 | GSM387791 | 40 | Intestinal | 4 | TP53+ | low-risk |
| GSE15459 | GSM387792 | 73 | Intestinal | 4 | TP53- | high-risk |
| GSE15459 | GSM387794 | 56 | Intestinal | 4 | EMT | high-risk |
| GSE15459 | GSM387795 | 29 | Diffuse | 3 | EMT | low-risk |
| GSE15459 | GSM387796 | 67 | Intestinal | 3 | TP53+ | low-risk |
| GSE15459 | GSM387800 | 69 | Mixed | 3 | TP53- | low-risk |
| GSE15459 | GSM387801 | 49 | Intestinal | 1 | TP53+ | low-risk |
| GSE15459 | GSM387802 | 78 | Mixed | 1 | TP53+ | low-risk |
| GSE15459 | GSM387803 | 88 | Intestinal | 4 | TP53- | low-risk |
| GSE15459 | GSM387804 | 72 | Diffuse | 3 | EMT | high-risk |
| GSE15459 | GSM387805 | 76 | Intestinal | 1 | TP53+ | low-risk |
| GSE15459 | GSM387806 | 76 | Diffuse | 3 | TP53+ | low-risk |
| GSE15459 | GSM387807 | 65 | Intestinal | 2 | TP53+ | low-risk |
| GSE15459 | GSM387808 | 73 | Intestinal | 4 | TP53- | low-risk |
| GSE15459 | GSM387809 | 82 | Diffuse | 3 | TP53- | low-risk |
| GSE15459 | GSM387810 | 73 | Mixed | 1 | TP53- | low-risk |
| GSE15459 | GSM387811 | 70 | Intestinal | 4 | MSI | high-risk |
| GSE15459 | GSM387812 | 72 | Intestinal | 3 | TP53+ | low-risk |
| GSE15459 | GSM387813 | 74 | Intestinal | 1 | MSI | high-risk |
| GSE15459 | GSM387814 | 27.6 | Diffuse | 4 | EMT | high-risk |
| GSE15459 | GSM387815 | 67.7 | Intestinal | 2 | TP53- | low-risk |
| GSE15459 | GSM387816 | 70.1 | Diffuse | 4 | EMT | high-risk |
| GSE15459 | GSM387817 | 64.3 | Intestinal | 1 | TP53- | low-risk |
| GSE15459 | GSM387818 | 52.6 | Intestinal | 2 | TP53+ | high-risk |
| GSE15459 | GSM387819 | 53.1 | Diffuse | 4 | EMT | low-risk |
| GSE15459 | GSM387820 | 52.7 | Intestinal | 4 | TP53- | low-risk |
| GSE15459 | GSM387821 | 53 | Intestinal | 1 | TP53- | low-risk |
| GSE15459 | GSM387822 | 78.3 | Intestinal | 3 | TP53+ | high-risk |
| GSE15459 | GSM387823 | 64.3 | Intestinal | 4 | EMT | high-risk |
| GSE15459 | GSM387824 | 71.3 | Intestinal | 2 | EMT | low-risk |
| GSE15459 | GSM387825 | 69.9 | Intestinal | 1 | TP53- | low-risk |
| GSE15459 | GSM387826 | 74.4 | Diffuse | 2 | EMT | high-risk |
| GSE15459 | GSM387827 | 67.4 | Intestinal | 1 | EMT | low-risk |
| GSE15459 | GSM387828 | 61.4 | Mixed | 3 | MSI | high-risk |
| GSE15459 | GSM387829 | 65.3 | Intestinal | 2 | EMT | low-risk |
| GSE15459 | GSM387830 | 67.9 | Diffuse | 3 | EMT | high-risk |
| GSE15459 | GSM387831 | 47.6 | Diffuse | 4 | TP53+ | low-risk |
| GSE15459 | GSM387832 | 31.8 | Diffuse | 3 | EMT | high-risk |
| GSE15459 | GSM387833 | 50.6 | Intestinal | 3 | EMT | high-risk |
| GSE15459 | GSM387834 | 67.6 | Diffuse | 2 | EMT | high-risk |
| GSE15459 | GSM387835 | 55.7 | Diffuse | 3 | EMT | high-risk |
| GSE15459 | GSM387836 | 52.8 | Diffuse | 3 | EMT | high-risk |
| GSE15459 | GSM387837 | 81.7 | Diffuse | 3 | EMT | high-risk |
| GSE15459 | GSM387838 | 62.4 | Intestinal | 3 | EMT | high-risk |
| GSE15459 | GSM387839 | 67.7 | Intestinal | 3 | TP53+ | high-risk |
| GSE15459 | GSM387840 | 55.9 | Diffuse | 1 | EMT | high-risk |
| GSE15459 | GSM387841 | 66.8 | Diffuse | 4 | EMT | high-risk |
| GSE15459 | GSM387842 | 76.8 | Intestinal | 4 | EMT | low-risk |
| GSE15459 | GSM387843 | 76.9 | Intestinal | 4 | TP53- | high-risk |
| GSE15459 | GSM387845 | 69.5 | Intestinal | 4 | TP53+ | low-risk |
| GSE15459 | GSM387846 | 85 | Intestinal | 3 | MSI | high-risk |
| GSE15459 | GSM387847 | 80 | Intestinal | 1 | EMT | low-risk |
| GSE15459 | GSM387848 | 58.2 | Intestinal | 3 | EMT | low-risk |
| GSE15459 | GSM387849 | 70.9 | Diffuse | 4 | EMT | high-risk |
| GSE15459 | GSM387850 | 59.9 | Diffuse | 4 | EMT | high-risk |
| GSE15459 | GSM387851 | 61.5 | Diffuse | 4 | EMT | high-risk |
| GSE15459 | GSM387852 | 61.7 | Mixed | 4 | EMT | low-risk |
| GSE15459 | GSM387853 | 65.2 | Intestinal | 4 | EMT | low-risk |
| GSE15459 | GSM387854 | 44.6 | Intestinal | 2 | EMT | high-risk |
| GSE15459 | GSM387855 | 71.3 | Intestinal | 2 | TP53- | high-risk |
| GSE15459 | GSM387856 | 80.4 | Intestinal | 3 | EMT | high-risk |
| GSE15459 | GSM387857 | 49.9 | Diffuse | 3 | EMT | high-risk |
| GSE15459 | GSM387858 | 39 | Diffuse | 4 | EMT | high-risk |
| GSE15459 | GSM387859 | 79.6 | Diffuse | 3 | EMT | high-risk |
| GSE15459 | GSM387860 | 66.8 | Intestinal | 4 | TP53+ | low-risk |
| GSE15459 | GSM387861 | 56.1 | Intestinal | 3 | TP53+ | high-risk |
| GSE15459 | GSM387862 | 64.1 | Diffuse | 4 | EMT | high-risk |
| GSE15459 | GSM387863 | 78.4 | Intestinal | 1 | EMT | high-risk |
| GSE15459 | GSM387864 | 67.5 | Diffuse | 3 | TP53- | low-risk |
| GSE15459 | GSM387865 | 64.8 | Diffuse | 4 | EMT | high-risk |
| GSE15459 | GSM387866 | 75.6 | Diffuse | 3 | EMT | low-risk |
| GSE15459 | GSM387867 | 48.9 | Diffuse | 2 | TP53+ | low-risk |
| GSE15459 | GSM387868 | 75.9 | Diffuse | 2 | EMT | high-risk |
| GSE15459 | GSM387869 | 49.2 | Mixed | 3 | EMT | high-risk |
| GSE15459 | GSM387870 | 56.3 | Intestinal | 4 | TP53+ | high-risk |
| GSE15459 | GSM387871 | 68.4 | Intestinal | 4 | TP53+ | low-risk |
| GSE15459 | GSM387872 | 62.8 | Intestinal | 1 | TP53- | low-risk |
| GSE15459 | GSM387873 | 64 | Diffuse | 3 | TP53- | low-risk |
| GSE15459 | GSM387874 | 81 | Intestinal | 4 | EMT | high-risk |
| GSE15459 | GSM387875 | 79.4 | Intestinal | 1 | EMT | low-risk |
| GSE15459 | GSM387876 | 45.6 | Diffuse | 2 | EMT | high-risk |
| GSE15459 | GSM387877 | 72.5 | Diffuse | 3 | EMT | high-risk |
| GSE15459 | GSM387878 | 63.3 | Diffuse | 2 | EMT | high-risk |
| GSE15459 | GSM387879 | 64.4 | Diffuse | 1 | TP53+ | low-risk |
| GSE15459 | GSM387880 | 66.2 | Diffuse | 1 | EMT | high-risk |
| GSE15459 | GSM387881 | 71.6 | Diffuse | 4 | EMT | high-risk |
| GSE15459 | GSM387882 | 84 | Intestinal | 3 | TP53- | high-risk |
| GSE15459 | GSM387883 | 76.4 | Intestinal | 2 | TP53- | low-risk |
| GSE15459 | GSM387884 | 87.7 | Diffuse | 2 | TP53+ | low-risk |
| GSE15459 | GSM387885 | 65.1 | Diffuse | 3 | TP53- | low-risk |
| GSE15459 | GSM387886 | 69.4 | Mixed | 2 | TP53+ | low-risk |
| GSE15459 | GSM387887 | 66.6 | Intestinal | 4 | TP53- | low-risk |
| GSE15459 | GSM387888 | 47.4 | Diffuse | 1 | EMT | high-risk |
| GSE15459 | GSM387889 | 77.1 | Mixed | 4 | EMT | high-risk |
| GSE15459 | GSM387890 | 62.8 | Diffuse | 3 | TP53- | high-risk |
| GSE15459 | GSM387891 | 53.2 | Diffuse | 1 | TP53- | low-risk |
| GSE15459 | GSM387892 | 79.5 | Intestinal | 4 | EMT | high-risk |
| GSE15459 | GSM387893 | 66.8 | Diffuse | 3 | EMT | low-risk |
| GSE15459 | GSM387894 | 84.5 | Intestinal | 1 | MSI | high-risk |
| GSE15459 | GSM387895 | 87.9 | Intestinal | 3 | TP53- | low-risk |
| GSE15459 | GSM387896 | 80.3 | Intestinal | 3 | TP53- | high-risk |
| GSE15459 | GSM387897 | 56.9 | Diffuse | 4 | TP53- | low-risk |
| GSE15459 | GSM387898 | 68.4 | Mixed | 2 | EMT | high-risk |
| GSE15459 | GSM387899 | 65 | Diffuse | 3 | EMT | high-risk |
| GSE15459 | GSM387900 | 68.4 | Intestinal | 1 | TP53- | low-risk |
| GSE15459 | GSM387901 | 64.1 | Intestinal | 1 | TP53+ | low-risk |
| GSE15459 | GSM387902 | 47.7 | Intestinal | 2 | TP53- | low-risk |
| GSE15459 | GSM387903 | 92.4 | Intestinal | 3 | TP53+ | low-risk |
| GSE15459 | GSM387904 | 75.4 | Mixed | 2 | TP53+ | low-risk |
| GSE15459 | GSM387905 | 51.2 | Diffuse | 4 | EMT | high-risk |
| GSE15459 | GSM387906 | 64.3 | Diffuse | 3 | EMT | low-risk |
| GSE15459 | GSM387907 | 69.5 | Mixed | 3 | TP53+ | low-risk |
| GSE15459 | GSM387908 | 62.3 | Intestinal | 3 | TP53- | high-risk |
| GSE15459 | GSM387909 | 56.5 | Diffuse | 4 | EMT | high-risk |
| GSE15459 | GSM387910 | 73.7 | Mixed | 4 | TP53- | high-risk |
| GSE15459 | GSM387911 | 43.2 | Mixed | 4 | EMT | low-risk |
| GSE15459 | GSM387912 | 38.1 | Diffuse | 3 | TP53+ | low-risk |
| GSE15459 | GSM387913 | 68.9 | Intestinal | 4 | TP53- | high-risk |
| GSE15459 | GSM387914 | 72.9 | Intestinal | 3 | TP53- | high-risk |
| GSE15459 | GSM387915 | 57.5 | Diffuse | 2 | EMT | high-risk |
| GSE15459 | GSM387916 | 80.8 | Intestinal | 1 | TP53- | low-risk |
| GSE15459 | GSM387917 | 58.9 | Intestinal | 4 | TP53+ | low-risk |
| GSE15459 | GSM387918 | 58.1 | Diffuse | 1 | TP53+ | low-risk |
| GSE15459 | GSM387919 | 74.1 | Intestinal | 4 | TP53- | high-risk |
| GSE15459 | GSM387920 | 59.1 | Intestinal | 3 | TP53- | low-risk |
| GSE15459 | GSM387921 | 80.2 | Intestinal | 4 | TP53+ | low-risk |
| GSE15459 | GSM387922 | 60.9 | Intestinal | 1 | MSI | low-risk |
| GSE15459 | GSM387923 | 70.2 | Intestinal | 3 | TP53- | high-risk |
| GSE15459 | GSM387924 | 45.7 | Diffuse | 4 | TP53- | high-risk |
| GSE15459 | GSM387925 | 33.4 | Diffuse | 2 | EMT | high-risk |
| GSE15459 | GSM387926 | 49.2 | Diffuse | 4 | TP53+ | high-risk |
| GSE15459 | GSM387927 | 48.6 | Diffuse | 4 | EMT | high-risk |
| GSE15459 | GSM387928 | 65.4 | Mixed | 2 | MSI | low-risk |
| GSE15459 | GSM387929 | 66.4 | Diffuse | 4 | EMT | high-risk |
| GSE15459 | GSM387930 | 63.6 | Intestinal | 3 | TP53+ | low-risk |
| GSE15459 | GSM387931 | 52.7 | Diffuse | 1 | EMT | high-risk |
| GSE15459 | GSM387932 | 66.5 | Intestinal | 4 | TP53- | low-risk |
| GSE15459 | GSM387933 | 68.8 | Diffuse | 4 | EMT | high-risk |
| GSE15459 | GSM387934 | 39 | Diffuse | 3 | TP53+ | high-risk |
| GSE15459 | GSM387935 | 23.4 | Intestinal | 4 | TP53- | low-risk |
| GSE15459 | GSM387936 | 67.8 | Mixed | 3 | EMT | low-risk |
| GSE15459 | GSM387938 | 69.8 | Diffuse | 4 | TP53- | low-risk |
| GSE15459 | GSM387939 | 46.7 | Diffuse | 1 | TP53- | low-risk |
| GSE15459 | GSM387940 | 75.9 | Diffuse | 3 | EMT | low-risk |
| GSE15459 | GSM387941 | 77.9 | Intestinal | 1 | TP53- | low-risk |
| GSE15459 | GSM387942 | 82.9 | Diffuse | 3 | EMT | high-risk |
| GSE15459 | GSM387943 | 67 | Diffuse | 4 | EMT | high-risk |
| GSE15459 | GSM387944 | 88 | Intestinal | 3 | TP53- | low-risk |
| GSE15459 | GSM387945 | 65 | Intestinal | 3 | TP53- | high-risk |
| GSE15459 | GSM387946 | 61.6 | Diffuse | 2 | EMT | low-risk |
| GSE15459 | GSM387947 | 65 | Intestinal | 4 | EMT | low-risk |
| GSE15459 | GSM387948 | 66 | Intestinal | 3 | TP53- | low-risk |
| GSE15459 | GSM387949 | 66.4 | Mixed | 3 | TP53+ | high-risk |
| GSE15459 | GSM387950 | 59.6 | Diffuse | 3 | EMT | high-risk |
| GSE15459 | GSM387951 | 69.6 | Intestinal | 4 | TP53- | low-risk |
| GSE15459 | GSM387952 | 68.1 | Intestinal | 3 | MSI | low-risk |
| GSE15459 | GSM387953 | 40.3 | Intestinal | 4 | MSI | high-risk |
| GSE15459 | GSM387954 | 69.1 | Intestinal | 3 | EMT | high-risk |
| GSE15459 | GSM387955 | 55.5 | Diffuse | 3 | TP53+ | high-risk |
| GSE15459 | GSM387956 | 25.2 | Diffuse | 3 | TP53- | high-risk |
| GSE15459 | GSM387957 | 73.3 | Diffuse | 3 | TP53- | low-risk |
| GSE15459 | GSM387958 | 69.2 | Diffuse | 1 | TP53+ | low-risk |
| GSE15459 | GSM387959 | 71.2 | Intestinal | 4 | EMT | low-risk |
| GSE15459 | GSM387960 | 59.2 | Intestinal | 3 | EMT | low-risk |
| GSE15459 | GSM387961 | 74.8 | Intestinal | 4 | EMT | high-risk |
| GSE15459 | GSM387962 | 64.2 | Intestinal | 3 | EMT | low-risk |
| GSE15459 | GSM387963 | 46.2 | Intestinal | 3 | TP53+ | low-risk |
| GSE15459 | GSM387964 | 53.9 | Intestinal | 4 | TP53+ | low-risk |
| GSE15459 | GSM387965 | 64.9 | Intestinal | 2 | TP53- | high-risk |
| GSE15459 | GSM387966 | 74.3 | Intestinal | 1 | MSI | low-risk |
| GSE15459 | GSM387967 | 56.4 | Diffuse | 3 | EMT | high-risk |
| GSE15459 | GSM387968 | 77.3 | Intestinal | 3 | TP53- | low-risk |
| GSE15459 | GSM387969 | 76.3 | Intestinal | 1 | EMT | low-risk |
| GSE15459 | GSM387970 | 40.7 | Intestinal | 3 | EMT | high-risk |
| GSE15459 | GSM387971 | 60.4 | Intestinal | 3 | TP53- | high-risk |
| GSE15459 | GSM387972 | 31.2 | Intestinal | 4 | EMT | high-risk |
| GSE15459 | GSM387973 | 79.5 | Intestinal | 3 | TP53- | low-risk |
| GSE15459 | GSM387974 | 72.3 | Intestinal | 2 | TP53- | low-risk |
| GSE15459 | GSM387975 | 72 | Intestinal | 3 | TP53- | high-risk |
| GSE15459 | GSM387976 | 71.6 | Intestinal | 2 | TP53- | low-risk |
| GSE15459 | GSM387977 | 50.5 | Intestinal | 3 | TP53- | low-risk |
| GSE15459 | GSM387978 | 58.5 | Diffuse | 2 | EMT | high-risk |
| GSE15459 | GSM387979 | 34.3 | Diffuse | 4 | TP53- | high-risk |
| GSE15459 | GSM387980 | 64.8 | Diffuse | 3 | EMT | high-risk |
| GSE15459 | GSM387981 | 64.6 | Intestinal | 3 | TP53- | high-risk |
| GSE15459 | GSM387982 | 67.7 | Diffuse | 2 | TP53- | low-risk |
| GSE15459 | GSM387983 | 70.8 | Diffuse | 3 | EMT | high-risk |
| GSE15459 | GSM387984 | 78.7 | Intestinal | 3 | MSI | low-risk |
| GSE15459 | GSM387985 | 70.9 | Intestinal | 2 | TP53- | low-risk |
| GSE15459 | GSM387986 | 79.4 | Mixed | 1 | TP53- | low-risk |
| GSE15459 | GSM387987 | 59.7 | Intestinal | 4 | TP53- | high-risk |
| GSE13861 | GSM348351 | 75 | NA | 2 | NA | low-risk |
| GSE13861 | GSM348352 | 64 | diffuse | 4 | NA | high-risk |
| GSE13861 | GSM348353 | 69 | intestinal | 1 | NA | low-risk |
| GSE13861 | GSM348354 | 51 | diffuse | 3 | NA | low-risk |
| GSE13861 | GSM348355 | 57 | NA | 4 | NA | high-risk |
| GSE13861 | GSM348356 | 62 | diffuse | 3 | NA | low-risk |
| GSE13861 | GSM348357 | 83 | mixed | 1 | NA | low-risk |
| GSE13861 | GSM348360 | 53 | intestinal | 3 | NA | low-risk |
| GSE13861 | GSM348361 | 74 | diffuse | 4 | NA | high-risk |
| GSE13861 | GSM348362 | 74 | NA | 4 | NA | high-risk |
| GSE13861 | GSM348363 | 66 | diffuse | 2 | NA | low-risk |
| GSE13861 | GSM348364 | 69 | intestinal | 3 | NA | low-risk |
| GSE13861 | GSM348365 | 63 | diffuse | 3 | NA | low-risk |
| GSE13861 | GSM348366 | 38 | diffuse | 4 | NA | low-risk |
| GSE13861 | GSM348367 | 58 | intestinal | 3 | NA | low-risk |
| GSE13861 | GSM348368 | 70 | diffuse | 2 | NA | low-risk |
| GSE13861 | GSM348369 | 44 | intestinal | 3 | NA | low-risk |
| GSE13861 | GSM348370 | 64 | diffuse | 4 | NA | high-risk |
| GSE13861 | GSM348371 | 76 | mixed | 1 | NA | low-risk |
| GSE13861 | GSM348372 | 78 | diffuse | 3 | NA | low-risk |
| GSE13861 | GSM348373 | 69 | diffuse | 3 | NA | high-risk |
| GSE13861 | GSM348375 | 67 | intestinal | 3 | NA | high-risk |
| GSE13861 | GSM348376 | 61 | diffuse | 1 | NA | high-risk |
| GSE13861 | GSM348377 | 69 | mixed | 2 | NA | low-risk |
| GSE13861 | GSM348378 | 34 | diffuse | 3 | NA | high-risk |
| GSE13861 | GSM348379 | 64 | intestinal | 1 | NA | low-risk |
| GSE13861 | GSM348380 | 37 | diffuse | 2 | NA | high-risk |
| GSE13861 | GSM348381 | 56 | intestinal | 3 | NA | high-risk |
| GSE13861 | GSM348382 | 76 | diffuse | 4 | NA | low-risk |
| GSE13861 | GSM348383 | 60 | diffuse | 4 | NA | low-risk |
| GSE13861 | GSM348384 | 69 | intestinal | 4 | NA | high-risk |
| GSE13861 | GSM348385 | 60 | mixed | 2 | NA | high-risk |
| GSE13861 | GSM348386 | 52 | mixed | 2 | NA | low-risk |
| GSE13861 | GSM348387 | 62 | diffuse | 1 | NA | high-risk |
| GSE13861 | GSM348388 | 32 | intestinal | 4 | NA | high-risk |
| GSE13861 | GSM348389 | 58 | NA | 2 | NA | low-risk |
| GSE13861 | GSM348390 | 68 | intestinal | 1 | NA | high-risk |
| GSE13861 | GSM348391 | 39 | intestinal | 3 | NA | high-risk |
| GSE13861 | GSM348392 | 57 | intestinal | 3 | NA | high-risk |
| GSE13861 | GSM348393 | 73 | mixed | 3 | NA | high-risk |
| GSE13861 | GSM348394 | 66 | diffuse | 1 | NA | low-risk |
| GSE13861 | GSM348395 | 67 | mixed | 3 | NA | low-risk |
| GSE13861 | GSM348396 | 66 | intestinal | 2 | NA | high-risk |
| GSE13861 | GSM348397 | 64 | diffuse | 4 | NA | high-risk |
| GSE13861 | GSM348398 | 66 | mixed | 1 | NA | low-risk |
| GSE13861 | GSM348399 | 52 | intestinal | 2 | NA | low-risk |
| GSE13861 | GSM348400 | 46 | diffuse | 4 | NA | low-risk |
| GSE13861 | GSM348401 | 76 | diffuse | 2 | NA | high-risk |
| GSE13861 | GSM348403 | 49 | diffuse | 4 | NA | high-risk |
| GSE13861 | GSM348405 | 62 | mixed | 3 | NA | low-risk |
| GSE13861 | GSM348406 | 47 | diffuse | 3 | NA | low-risk |
| GSE13861 | GSM348407 | 61 | mixed | 3 | NA | high-risk |
| GSE13861 | GSM348408 | 64 | diffuse | 1 | NA | low-risk |
| GSE13861 | GSM348409 | 51 | diffuse | 2 | NA | high-risk |
| GSE13861 | GSM348410 | 80 | mixed | 3 | NA | high-risk |
| GSE13861 | GSM348411 | 43 | intestinal | 4 | NA | high-risk |
| GSE13861 | GSM348412 | 60 | intestinal | 3 | NA | high-risk |
| GSE13861 | GSM348413 | 55 | diffuse | 3 | NA | low-risk |
| GSE13861 | GSM348414 | 72 | intestinal | 3 | NA | high-risk |
| GSE13861 | GSM348415 | 56 | diffuse | 3 | NA | high-risk |
| GSE13861 | GSM348416 | 52 | mixed | 1 | NA | low-risk |
| GSE13861 | GSM348418 | 71 | diffuse | 3 | NA | low-risk |
| GSE13861 | GSM348419 | 58 | diffuse | 1 | NA | high-risk |
| GSE13861 | GSM348420 | 67 | diffuse | 4 | NA | high-risk |
| GSE13861 | GSM348421 | 64 | intestinal | 4 | NA | high-risk |
| GSE84437 | GSM2235556 | 61 | NA | NA | NA | high-risk |
| GSE84437 | GSM2235557 | 47 | NA | NA | NA | low-risk |
| GSE84437 | GSM2235558 | 72 | NA | NA | NA | high-risk |
| GSE84437 | GSM2235559 | 78 | NA | NA | NA | high-risk |
| GSE84437 | GSM2235560 | 58 | NA | NA | NA | high-risk |
| GSE84437 | GSM2235561 | 33 | NA | NA | NA | high-risk |
| GSE84437 | GSM2235562 | 81 | NA | NA | NA | low-risk |
| GSE84437 | GSM2235563 | 60 | NA | NA | NA | high-risk |
| GSE84437 | GSM2235564 | 61 | NA | NA | NA | low-risk |
| GSE84437 | GSM2235565 | 49 | NA | NA | NA | high-risk |
| GSE84437 | GSM2235566 | 44 | NA | NA | NA | high-risk |
| GSE84437 | GSM2235567 | 51 | NA | NA | NA | high-risk |
| GSE84437 | GSM2235568 | 58 | NA | NA | NA | high-risk |
| GSE84437 | GSM2235569 | 79 | NA | NA | NA | high-risk |
| GSE84437 | GSM2235570 | 77 | NA | NA | NA | high-risk |
| GSE84437 | GSM2235571 | 85 | NA | NA | NA | high-risk |
| GSE84437 | GSM2235572 | 68 | NA | NA | NA | high-risk |
| GSE84437 | GSM2235573 | 52 | NA | NA | NA | high-risk |
| GSE84437 | GSM2235574 | 72 | NA | NA | NA | high-risk |
| GSE84437 | GSM2235575 | 59 | NA | NA | NA | low-risk |
| GSE84437 | GSM2235576 | 78 | NA | NA | NA | low-risk |
| GSE84437 | GSM2235577 | 32 | NA | NA | NA | high-risk |
| GSE84437 | GSM2235578 | 68 | NA | NA | NA | low-risk |
| GSE84437 | GSM2235579 | 74 | NA | NA | NA | high-risk |
| GSE84437 | GSM2235580 | 61 | NA | NA | NA | high-risk |
| GSE84437 | GSM2235582 | 65 | NA | NA | NA | high-risk |
| GSE84437 | GSM2235584 | 71 | NA | NA | NA | high-risk |
| GSE84437 | GSM2235585 | 71 | NA | NA | NA | low-risk |
| GSE84437 | GSM2235586 | 71 | NA | NA | NA | high-risk |
| GSE84437 | GSM2235587 | 72 | NA | NA | NA | high-risk |
| GSE84437 | GSM2235588 | 56 | NA | NA | NA | high-risk |
| GSE84437 | GSM2235589 | 66 | NA | NA | NA | low-risk |
| GSE84437 | GSM2235593 | 56 | NA | NA | NA | low-risk |
| GSE84437 | GSM2235595 | 74 | NA | NA | NA | high-risk |
| GSE84437 | GSM2235596 | 63 | NA | NA | NA | low-risk |
| GSE84437 | GSM2235597 | 68 | NA | NA | NA | low-risk |
| GSE84437 | GSM2235598 | 74 | NA | NA | NA | high-risk |
| GSE84437 | GSM2235599 | 62 | NA | NA | NA | low-risk |
| GSE84437 | GSM2235600 | 53 | NA | NA | NA | low-risk |
| GSE84437 | GSM2235601 | 79 | NA | NA | NA | high-risk |
| GSE84437 | GSM2235602 | 61 | NA | NA | NA | high-risk |
| GSE84437 | GSM2235603 | 60 | NA | NA | NA | high-risk |
| GSE84437 | GSM2235604 | 75 | NA | NA | NA | high-risk |
| GSE84437 | GSM2235605 | 54 | NA | NA | NA | low-risk |
| GSE84437 | GSM2235606 | 38 | NA | NA | NA | high-risk |
| GSE84437 | GSM2235607 | 65 | NA | NA | NA | high-risk |
| GSE84437 | GSM2235608 | 55 | NA | NA | NA | low-risk |
| GSE84437 | GSM2235609 | 73 | NA | NA | NA | high-risk |
| GSE84437 | GSM2235610 | 76 | NA | NA | NA | high-risk |
| GSE84437 | GSM2235611 | 78 | NA | NA | NA | high-risk |
| GSE84437 | GSM2235612 | 69 | NA | NA | NA | high-risk |
| GSE84437 | GSM2235613 | 68 | NA | NA | NA | low-risk |
| GSE84437 | GSM2235614 | 27 | NA | NA | NA | low-risk |
| GSE84437 | GSM2235615 | 68 | NA | NA | NA | high-risk |
| GSE84437 | GSM2235616 | 73 | NA | NA | NA | high-risk |
| GSE84437 | GSM2235617 | 54 | NA | NA | NA | low-risk |
| GSE84437 | GSM2235618 | 73 | NA | NA | NA | low-risk |
| GSE84437 | GSM2235619 | 55 | NA | NA | NA | high-risk |
| GSE84437 | GSM2235620 | 65 | NA | NA | NA | low-risk |
| GSE84437 | GSM2235621 | 63 | NA | NA | NA | low-risk |
| GSE84437 | GSM2235622 | 50 | NA | NA | NA | high-risk |
| GSE84437 | GSM2235623 | 56 | NA | NA | NA | low-risk |
| GSE84437 | GSM2235624 | 59 | NA | NA | NA | low-risk |
| GSE84437 | GSM2235625 | 53 | NA | NA | NA | high-risk |
| GSE84437 | GSM2235626 | 46 | NA | NA | NA | high-risk |
| GSE84437 | GSM2235627 | 68 | NA | NA | NA | low-risk |
| GSE84437 | GSM2235628 | 43 | NA | NA | NA | high-risk |
| GSE84437 | GSM2235629 | 78 | NA | NA | NA | high-risk |
| GSE84437 | GSM2235630 | 65 | NA | NA | NA | high-risk |
| GSE84437 | GSM2235631 | 66 | NA | NA | NA | high-risk |
| GSE84437 | GSM2235632 | 60 | NA | NA | NA | low-risk |
| GSE84437 | GSM2235633 | 63 | NA | NA | NA | high-risk |
| GSE84437 | GSM2235634 | 64 | NA | NA | NA | low-risk |
| GSE84437 | GSM2235635 | 73 | NA | NA | NA | low-risk |
| GSE84437 | GSM2235636 | 32 | NA | NA | NA | high-risk |
| GSE84437 | GSM2235637 | 75 | NA | NA | NA | low-risk |
| GSE84437 | GSM2235695 | 42 | NA | NA | NA | low-risk |
| GSE84437 | GSM2235696 | 60 | NA | NA | NA | low-risk |
| GSE84437 | GSM2235697 | 49 | NA | NA | NA | low-risk |
| GSE84437 | GSM2235698 | 34 | NA | NA | NA | low-risk |
| GSE84437 | GSM2235699 | 68 | NA | NA | NA | low-risk |
| GSE84437 | GSM2235700 | 59 | NA | NA | NA | high-risk |
| GSE84437 | GSM2235701 | 61 | NA | NA | NA | low-risk |
| GSE84437 | GSM2235702 | 74 | NA | NA | NA | high-risk |
| GSE84437 | GSM2235703 | 35 | NA | NA | NA | high-risk |
| GSE84437 | GSM2235704 | 53 | NA | NA | NA | high-risk |
| GSE84437 | GSM2235705 | 67 | NA | NA | NA | low-risk |
| GSE84437 | GSM2235706 | 71 | NA | NA | NA | low-risk |
| GSE84437 | GSM2235707 | 63 | NA | NA | NA | low-risk |
| GSE84437 | GSM2235708 | 66 | NA | NA | NA | low-risk |
| GSE84437 | GSM2235709 | 54 | NA | NA | NA | low-risk |
| GSE84437 | GSM2235710 | 54 | NA | NA | NA | high-risk |
| GSE84437 | GSM2235711 | 65 | NA | NA | NA | low-risk |
| GSE84437 | GSM2235712 | 70 | NA | NA | NA | low-risk |
| GSE84437 | GSM2235713 | 59 | NA | NA | NA | high-risk |
| GSE84437 | GSM2235714 | 61 | NA | NA | NA | low-risk |
| GSE84437 | GSM2235715 | 58 | NA | NA | NA | low-risk |
| GSE84437 | GSM2235716 | 46 | NA | NA | NA | high-risk |
| GSE84437 | GSM2235717 | 43 | NA | NA | NA | high-risk |
| GSE84437 | GSM2235718 | 46 | NA | NA | NA | high-risk |
| GSE84437 | GSM2235719 | 68 | NA | NA | NA | low-risk |
| GSE84437 | GSM2235720 | 56 | NA | NA | NA | high-risk |
| GSE84437 | GSM2235721 | 41 | NA | NA | NA | low-risk |
| GSE84437 | GSM2235722 | 63 | NA | NA | NA | high-risk |
| GSE84437 | GSM2235723 | 52 | NA | NA | NA | high-risk |
| GSE84437 | GSM2235724 | 74 | NA | NA | NA | high-risk |
| GSE84437 | GSM2235725 | 65 | NA | NA | NA | high-risk |
| GSE84437 | GSM2235726 | 68 | NA | NA | NA | high-risk |
| GSE84437 | GSM2235727 | 71 | NA | NA | NA | low-risk |
| GSE84437 | GSM2235728 | 40 | NA | NA | NA | high-risk |
| GSE84437 | GSM2235729 | 67 | NA | NA | NA | high-risk |
| GSE84437 | GSM2235730 | 68 | NA | NA | NA | low-risk |
| GSE84437 | GSM2235731 | 63 | NA | NA | NA | low-risk |
| GSE84437 | GSM2235732 | 74 | NA | NA | NA | low-risk |
| GSE84437 | GSM2235733 | 72 | NA | NA | NA | high-risk |
| GSE84437 | GSM2235734 | 45 | NA | NA | NA | low-risk |
| GSE84437 | GSM2235735 | 53 | NA | NA | NA | low-risk |
| GSE84437 | GSM2235736 | 61 | NA | NA | NA | low-risk |
| GSE84437 | GSM2235737 | 51 | NA | NA | NA | low-risk |
| GSE84437 | GSM2235738 | 59 | NA | NA | NA | low-risk |
| GSE84437 | GSM2235739 | 66 | NA | NA | NA | high-risk |
| GSE84437 | GSM2235740 | 69 | NA | NA | NA | low-risk |
| GSE84437 | GSM2235741 | 49 | NA | NA | NA | high-risk |
| GSE84437 | GSM2235742 | 62 | NA | NA | NA | high-risk |
| GSE84437 | GSM2235743 | 64 | NA | NA | NA | low-risk |
| GSE84437 | GSM2235744 | 38 | NA | NA | NA | low-risk |
| GSE84437 | GSM2235745 | 53 | NA | NA | NA | high-risk |
| GSE84437 | GSM2235746 | 53 | NA | NA | NA | low-risk |
| GSE84437 | GSM2235747 | 53 | NA | NA | NA | high-risk |
| GSE84437 | GSM2235748 | 80 | NA | NA | NA | high-risk |
| GSE84437 | GSM2235749 | 65 | NA | NA | NA | high-risk |
| GSE84437 | GSM2235750 | 70 | NA | NA | NA | high-risk |
| GSE84437 | GSM2235751 | 40 | NA | NA | NA | low-risk |
| GSE84437 | GSM2235752 | 42 | NA | NA | NA | low-risk |
| GSE84437 | GSM2235753 | 72 | NA | NA | NA | high-risk |
| GSE84437 | GSM2235754 | 37 | NA | NA | NA | high-risk |
| GSE84437 | GSM2235755 | 62 | NA | NA | NA | high-risk |
| GSE84437 | GSM2235756 | 48 | NA | NA | NA | low-risk |
| GSE84437 | GSM2235757 | 44 | NA | NA | NA | low-risk |
| GSE84437 | GSM2235758 | 42 | NA | NA | NA | high-risk |
| GSE84437 | GSM2235759 | 54 | NA | NA | NA | high-risk |
| GSE84437 | GSM2235760 | 64 | NA | NA | NA | high-risk |
| GSE84437 | GSM2235761 | 63 | NA | NA | NA | high-risk |
| GSE84437 | GSM2235762 | 65 | NA | NA | NA | high-risk |
| GSE84437 | GSM2235763 | 74 | NA | NA | NA | high-risk |
| GSE84437 | GSM2235764 | 58 | NA | NA | NA | low-risk |
| GSE84437 | GSM2235765 | 68 | NA | NA | NA | high-risk |
| GSE84437 | GSM2235766 | 61 | NA | NA | NA | low-risk |
| GSE84437 | GSM2235767 | 53 | NA | NA | NA | high-risk |
| GSE84437 | GSM2235768 | 46 | NA | NA | NA | low-risk |
| GSE84437 | GSM2235769 | 64 | NA | NA | NA | low-risk |
| GSE84437 | GSM2235770 | 85 | NA | NA | NA | high-risk |
| GSE84437 | GSM2235771 | 53 | NA | NA | NA | high-risk |
| GSE84437 | GSM2235772 | 60 | NA | NA | NA | high-risk |
| GSE84437 | GSM2235773 | 30 | NA | NA | NA | high-risk |
| GSE84437 | GSM2235774 | 47 | NA | NA | NA | high-risk |
| GSE84437 | GSM2235775 | 60 | NA | NA | NA | high-risk |
| GSE84437 | GSM2235776 | 80 | NA | NA | NA | high-risk |
| GSE84437 | GSM2235777 | 49 | NA | NA | NA | high-risk |
| GSE84437 | GSM2235778 | 61 | NA | NA | NA | high-risk |
| GSE84437 | GSM2235779 | 63 | NA | NA | NA | low-risk |
| GSE84437 | GSM2235780 | 75 | NA | NA | NA | low-risk |
| GSE84437 | GSM2235781 | 56 | NA | NA | NA | low-risk |
| GSE84437 | GSM2235782 | 62 | NA | NA | NA | high-risk |
| GSE84437 | GSM2235783 | 71 | NA | NA | NA | high-risk |
| GSE84437 | GSM2235784 | 80 | NA | NA | NA | low-risk |
| GSE84437 | GSM2235785 | 64 | NA | NA | NA | low-risk |
| GSE84437 | GSM2235786 | 50 | NA | NA | NA | high-risk |
| GSE84437 | GSM2235787 | 58 | NA | NA | NA | high-risk |
| GSE84437 | GSM2235788 | 56 | NA | NA | NA | low-risk |
| GSE84437 | GSM2235789 | 64 | NA | NA | NA | low-risk |
| GSE84437 | GSM2235790 | 67 | NA | NA | NA | low-risk |
| GSE84437 | GSM2235791 | 36 | NA | NA | NA | high-risk |
| GSE84437 | GSM2235792 | 60 | NA | NA | NA | high-risk |
| GSE84437 | GSM2235793 | 83 | NA | NA | NA | high-risk |
| GSE84437 | GSM2235794 | 55 | NA | NA | NA | low-risk |
| GSE84437 | GSM2235795 | 73 | NA | NA | NA | high-risk |
| GSE84437 | GSM2235796 | 62 | NA | NA | NA | high-risk |
| GSE84437 | GSM2235797 | 79 | NA | NA | NA | low-risk |
| GSE84437 | GSM2235798 | 76 | NA | NA | NA | low-risk |
| GSE84437 | GSM2235799 | 62 | NA | NA | NA | low-risk |
| GSE84437 | GSM2235800 | 62 | NA | NA | NA | high-risk |
| GSE84437 | GSM2235801 | 50 | NA | NA | NA | high-risk |
| GSE84437 | GSM2235802 | 60 | NA | NA | NA | low-risk |
| GSE84437 | GSM2235803 | 46 | NA | NA | NA | low-risk |
| GSE84437 | GSM2235804 | 64 | NA | NA | NA | high-risk |
| GSE84437 | GSM2235805 | 61 | NA | NA | NA | high-risk |
| GSE84437 | GSM2235806 | 55 | NA | NA | NA | low-risk |
| GSE84437 | GSM2235807 | 70 | NA | NA | NA | low-risk |
| GSE84437 | GSM2235808 | 59 | NA | NA | NA | low-risk |
| GSE84437 | GSM2235809 | 58 | NA | NA | NA | high-risk |
| GSE84437 | GSM2235810 | 54 | NA | NA | NA | low-risk |
| GSE84437 | GSM2235811 | 55 | NA | NA | NA | high-risk |
| GSE84437 | GSM2235812 | 68 | NA | NA | NA | low-risk |
| GSE84437 | GSM2235813 | 64 | NA | NA | NA | low-risk |
| GSE84437 | GSM2235814 | 66 | NA | NA | NA | high-risk |
| GSE84437 | GSM2235815 | 71 | NA | NA | NA | high-risk |
| GSE84437 | GSM2235816 | 48 | NA | NA | NA | low-risk |
| GSE84437 | GSM2235817 | 68 | NA | NA | NA | low-risk |
| GSE84437 | GSM2235818 | 74 | NA | NA | NA | low-risk |
| GSE84437 | GSM2235819 | 68 | NA | NA | NA | high-risk |
| GSE84437 | GSM2235820 | 75 | NA | NA | NA | high-risk |
| GSE84437 | GSM2235821 | 67 | NA | NA | NA | low-risk |
| GSE84437 | GSM2235822 | 55 | NA | NA | NA | low-risk |
| GSE84437 | GSM2235823 | 32 | NA | NA | NA | high-risk |
| GSE84437 | GSM2235824 | 49 | NA | NA | NA | high-risk |
| GSE84437 | GSM2235825 | 69 | NA | NA | NA | high-risk |
| GSE84437 | GSM2235826 | 79 | NA | NA | NA | low-risk |
| GSE84437 | GSM2235827 | 47 | NA | NA | NA | high-risk |
| GSE84437 | GSM2235828 | 55 | NA | NA | NA | low-risk |
| GSE84437 | GSM2235829 | 52 | NA | NA | NA | low-risk |
| GSE84437 | GSM2235830 | 75 | NA | NA | NA | low-risk |
| GSE84437 | GSM2235831 | 81 | NA | NA | NA | low-risk |
| GSE84437 | GSM2235837 | 56 | NA | NA | NA | low-risk |
| GSE84437 | GSM2235838 | 65 | NA | NA | NA | high-risk |
| GSE84437 | GSM2235839 | 63 | NA | NA | NA | high-risk |
| GSE84437 | GSM2235840 | 71 | NA | NA | NA | low-risk |
| GSE84437 | GSM2235841 | 84 | NA | NA | NA | low-risk |
| GSE84437 | GSM2235842 | 72 | NA | NA | NA | high-risk |
| GSE84437 | GSM2235843 | 60 | NA | NA | NA | low-risk |
| GSE84437 | GSM2235844 | 54 | NA | NA | NA | low-risk |
| GSE84437 | GSM2235845 | 51 | NA | NA | NA | low-risk |
| GSE84437 | GSM2235846 | 67 | NA | NA | NA | low-risk |
| GSE84437 | GSM2235847 | 53 | NA | NA | NA | high-risk |
| GSE84437 | GSM2235848 | 52 | NA | NA | NA | high-risk |
| GSE84437 | GSM2235849 | 61 | NA | NA | NA | high-risk |
| GSE84437 | GSM2235850 | 51 | NA | NA | NA | high-risk |
| GSE84437 | GSM2235851 | 81 | NA | NA | NA | low-risk |
| GSE84437 | GSM2235852 | 69 | NA | NA | NA | high-risk |
| GSE84437 | GSM2235853 | 58 | NA | NA | NA | low-risk |
| GSE84437 | GSM2235854 | 66 | NA | NA | NA | high-risk |
| GSE84437 | GSM2235855 | 68 | NA | NA | NA | low-risk |
| GSE84437 | GSM2235862 | 66 | NA | NA | NA | low-risk |
| GSE84437 | GSM2235863 | 67 | NA | NA | NA | high-risk |
| GSE84437 | GSM2235864 | 54 | NA | NA | NA | high-risk |
| GSE84437 | GSM2235865 | 44 | NA | NA | NA | low-risk |
| GSE84437 | GSM2235866 | 79 | NA | NA | NA | high-risk |
| GSE84437 | GSM2235867 | 69 | NA | NA | NA | high-risk |
| GSE84437 | GSM2235878 | 51 | NA | NA | NA | low-risk |
| GSE84437 | GSM2235879 | 68 | NA | NA | NA | high-risk |
| GSE84437 | GSM2235880 | 67 | NA | NA | NA | low-risk |
| GSE84437 | GSM2235881 | 72 | NA | NA | NA | low-risk |
| GSE84437 | GSM2235882 | 55 | NA | NA | NA | low-risk |
| GSE84437 | GSM2235883 | 66 | NA | NA | NA | high-risk |
| GSE84437 | GSM2235884 | 44 | NA | NA | NA | low-risk |
| GSE84437 | GSM2235885 | 73 | NA | NA | NA | high-risk |
| GSE84437 | GSM2235886 | 76 | NA | NA | NA | low-risk |
| GSE84437 | GSM2235887 | 81 | NA | NA | NA | high-risk |
| GSE84437 | GSM2235888 | 36 | NA | NA | NA | low-risk |
| GSE84437 | GSM2235889 | 66 | NA | NA | NA | low-risk |
| GSE84437 | GSM2235899 | 48 | NA | NA | NA | low-risk |
| GSE84437 | GSM2235900 | 46 | NA | NA | NA | low-risk |
| GSE84437 | GSM2235901 | 66 | NA | NA | NA | low-risk |
| GSE84437 | GSM2235902 | 34 | NA | NA | NA | high-risk |
| GSE84437 | GSM2235903 | 50 | NA | NA | NA | high-risk |
| GSE84437 | GSM2235904 | 62 | NA | NA | NA | high-risk |
| GSE84437 | GSM2235905 | 63 | NA | NA | NA | high-risk |
| GSE84437 | GSM2235906 | 70 | NA | NA | NA | low-risk |
| GSE84437 | GSM2235907 | 77 | NA | NA | NA | low-risk |
| GSE84437 | GSM2235908 | 32 | NA | NA | NA | high-risk |
| GSE84437 | GSM2235909 | 63 | NA | NA | NA | high-risk |
| GSE84437 | GSM2235910 | 56 | NA | NA | NA | high-risk |
| GSE84437 | GSM2235921 | 57 | NA | NA | NA | low-risk |
| GSE84437 | GSM2235922 | 68 | NA | NA | NA | high-risk |
| GSE84437 | GSM2235923 | 77 | NA | NA | NA | low-risk |
| GSE84437 | GSM2235924 | 54 | NA | NA | NA | high-risk |
| GSE84437 | GSM2235925 | 64 | NA | NA | NA | low-risk |
| GSE84437 | GSM2235926 | 47 | NA | NA | NA | high-risk |
| GSE84437 | GSM2235927 | 65 | NA | NA | NA | low-risk |
| GSE84437 | GSM2235928 | 56 | NA | NA | NA | low-risk |
| GSE84437 | GSM2235929 | 74 | NA | NA | NA | high-risk |
| GSE84437 | GSM2235930 | 63 | NA | NA | NA | high-risk |
| GSE84437 | GSM2235931 | 34 | NA | NA | NA | high-risk |
| GSE84437 | GSM2235932 | 71 | NA | NA | NA | low-risk |
| GSE84437 | GSM2235933 | 57 | NA | NA | NA | high-risk |
| GSE84437 | GSM2235934 | 63 | NA | NA | NA | low-risk |
| GSE84437 | GSM2235935 | 65 | NA | NA | NA | high-risk |
| GSE84437 | GSM2235936 | 63 | NA | NA | NA | low-risk |
| GSE84437 | GSM2235937 | 54 | NA | NA | NA | low-risk |
| GSE84437 | GSM2235938 | 62 | NA | NA | NA | low-risk |
| GSE84437 | GSM2235939 | 42 | NA | NA | NA | high-risk |
| GSE84437 | GSM2235940 | 63 | NA | NA | NA | low-risk |
| GSE84437 | GSM2235941 | 36 | NA | NA | NA | low-risk |
| GSE84437 | GSM2235942 | 67 | NA | NA | NA | low-risk |
| GSE84437 | GSM2235943 | 65 | NA | NA | NA | high-risk |
| GSE84437 | GSM2235944 | 47 | NA | NA | NA | high-risk |
| GSE84437 | GSM2235945 | 59 | NA | NA | NA | high-risk |
| GSE84437 | GSM2235946 | 55 | NA | NA | NA | low-risk |
| GSE84437 | GSM2235947 | 62 | NA | NA | NA | low-risk |
| GSE84437 | GSM2235948 | 36 | NA | NA | NA | high-risk |
| GSE84437 | GSM2235949 | 43 | NA | NA | NA | low-risk |
| GSE84437 | GSM2235950 | 45 | NA | NA | NA | high-risk |
| GSE84437 | GSM2235951 | 61 | NA | NA | NA | high-risk |
| GSE84437 | GSM2235952 | 56 | NA | NA | NA | low-risk |
| GSE84437 | GSM2235953 | 58 | NA | NA | NA | high-risk |
| GSE84437 | GSM2235954 | 39 | NA | NA | NA | low-risk |
| GSE84437 | GSM2235955 | 48 | NA | NA | NA | high-risk |
| GSE84437 | GSM2235956 | 68 | NA | NA | NA | low-risk |
| GSE84437 | GSM2235957 | 69 | NA | NA | NA | high-risk |
| GSE84437 | GSM2235958 | 55 | NA | NA | NA | high-risk |
| GSE84437 | GSM2235959 | 61 | NA | NA | NA | high-risk |
| GSE84437 | GSM2235960 | 66 | NA | NA | NA | low-risk |
| GSE84437 | GSM2235961 | 55 | NA | NA | NA | high-risk |
| GSE84437 | GSM2235962 | 64 | NA | NA | NA | high-risk |
| GSE84437 | GSM2235963 | 58 | NA | NA | NA | high-risk |
| GSE84437 | GSM2235964 | 64 | NA | NA | NA | low-risk |
| GSE84437 | GSM2235965 | 58 | NA | NA | NA | high-risk |
| GSE84437 | GSM2235966 | 40 | NA | NA | NA | low-risk |
| GSE84437 | GSM2235967 | 61 | NA | NA | NA | low-risk |
| GSE84437 | GSM2235968 | 52 | NA | NA | NA | high-risk |
| GSE84437 | GSM2235969 | 72 | NA | NA | NA | low-risk |
| GSE84437 | GSM2235970 | 66 | NA | NA | NA | high-risk |
| GSE84437 | GSM2235971 | 45 | NA | NA | NA | high-risk |
| GSE84437 | GSM2235972 | 67 | NA | NA | NA | low-risk |
| GSE84437 | GSM2235973 | 59 | NA | NA | NA | low-risk |
| GSE84437 | GSM2235974 | 63 | NA | NA | NA | low-risk |
| GSE84437 | GSM2235975 | 59 | NA | NA | NA | low-risk |
| GSE84437 | GSM2235976 | 67 | NA | NA | NA | low-risk |
| GSE84437 | GSM2235977 | 72 | NA | NA | NA | high-risk |
| GSE84437 | GSM2235978 | 65 | NA | NA | NA | low-risk |
| GSE84437 | GSM2235979 | 54 | NA | NA | NA | high-risk |
| GSE84437 | GSM2235980 | 66 | NA | NA | NA | high-risk |
| GSE84437 | GSM2235981 | 69 | NA | NA | NA | low-risk |
| GSE84437 | GSM2235982 | 51 | NA | NA | NA | high-risk |
| GSE84437 | GSM2235983 | 46 | NA | NA | NA | high-risk |
| GSE84437 | GSM2235984 | 69 | NA | NA | NA | high-risk |
| GSE84437 | GSM2235985 | 69 | NA | NA | NA | high-risk |
| GSE84437 | GSM2235986 | 53 | NA | NA | NA | low-risk |
| GSE84437 | GSM2235987 | 69 | NA | NA | NA | low-risk |
| GSE84437 | GSM2235988 | 54 | NA | NA | NA | low-risk |
| GSE84437 | GSM2235991 | 77 | NA | NA | NA | high-risk |
| GSE84437 | GSM2235992 | 59 | NA | NA | NA | high-risk |
| GSE84437 | GSM2235993 | 27 | NA | NA | NA | low-risk |
| GSE84437 | GSM2235994 | 42 | NA | NA | NA | high-risk |
| GSE84437 | GSM2235995 | 57 | NA | NA | NA | low-risk |
| GSE84437 | GSM2235996 | 53 | NA | NA | NA | low-risk |
| GSE84437 | GSM2235997 | 74 | NA | NA | NA | high-risk |
| GSE84437 | GSM2235998 | 68 | NA | NA | NA | low-risk |
| GSE84437 | GSM2235999 | 60 | NA | NA | NA | high-risk |
| GSE84437 | GSM2236000 | 64 | NA | NA | NA | high-risk |
| GSE84437 | GSM2236001 | 48 | NA | NA | NA | low-risk |
| GSE84437 | GSM2236002 | 68 | NA | NA | NA | low-risk |
| GSE84437 | GSM2236003 | 55 | NA | NA | NA | high-risk |
| GSE84437 | GSM2236004 | 66 | NA | NA | NA | low-risk |
| GSE84437 | GSM2236005 | 72 | NA | NA | NA | high-risk |
| GSE84437 | GSM2236006 | 42 | NA | NA | NA | high-risk |
| GSE84437 | GSM2236007 | 76 | NA | NA | NA | high-risk |
| GSE84437 | GSM2236008 | 64 | NA | NA | NA | high-risk |
| GSE84437 | GSM2236009 | 66 | NA | NA | NA | high-risk |
| GSE84437 | GSM2236010 | 61 | NA | NA | NA | low-risk |
| GSE84437 | GSM2236011 | 64 | NA | NA | NA | high-risk |
| GSE84437 | GSM2236012 | 33 | NA | NA | NA | low-risk |
| GSE84437 | GSM2236013 | 67 | NA | NA | NA | low-risk |
| GSE84437 | GSM2236014 | 68 | NA | NA | NA | low-risk |
| GSE84437 | GSM2236015 | 66 | NA | NA | NA | high-risk |
| GSE84437 | GSM2236016 | 53 | NA | NA | NA | low-risk |
| GSE84437 | GSM2236017 | 61 | NA | NA | NA | high-risk |
| GSE84437 | GSM2236018 | 36 | NA | NA | NA | low-risk |
| GSE84437 | GSM2236019 | 77 | NA | NA | NA | low-risk |
| GSE84437 | GSM2236020 | 70 | NA | NA | NA | low-risk |
| GSE84437 | GSM2236021 | 32 | NA | NA | NA | high-risk |
| GSE84437 | GSM2236022 | 53 | NA | NA | NA | low-risk |
| GSE84437 | GSM2236023 | 61 | NA | NA | NA | low-risk |
| GSE84437 | GSM2236024 | 54 | NA | NA | NA | low-risk |
| GSE84437 | GSM2236025 | 68 | NA | NA | NA | high-risk |
| GSE84437 | GSM2236026 | 65 | NA | NA | NA | high-risk |
| GSE84437 | GSM2236027 | 80 | NA | NA | NA | high-risk |
| GSE84437 | GSM2236028 | 68 | NA | NA | NA | low-risk |
| GSE84437 | GSM2236029 | 44 | NA | NA | NA | low-risk |
| GSE84437 | GSM2236030 | 63 | NA | NA | NA | low-risk |
| GSE84437 | GSM2236031 | 52 | NA | NA | NA | high-risk |
| GSE84437 | GSM2236032 | 44 | NA | NA | NA | low-risk |
| GSE84437 | GSM2236033 | 69 | NA | NA | NA | high-risk |
| GSE84437 | GSM2236034 | 55 | NA | NA | NA | low-risk |
| GSE84437 | GSM2236036 | 62 | NA | NA | NA | low-risk |
| GSE84437 | GSM2236037 | 64 | NA | NA | NA | high-risk |
| GSE84437 | GSM2236038 | 65 | NA | NA | NA | low-risk |
| GSE84437 | GSM2236039 | 56 | NA | NA | NA | low-risk |
| GSE84437 | GSM2236040 | 71 | NA | NA | NA | low-risk |
| GSE84437 | GSM2236041 | 50 | NA | NA | NA | low-risk |
| GSE84437 | GSM2236042 | 69 | NA | NA | NA | low-risk |
| GSE84437 | GSM2236043 | 48 | NA | NA | NA | low-risk |
| GSE84437 | GSM2236044 | 48 | NA | NA | NA | low-risk |
| GSE84437 | GSM2236045 | 63 | NA | NA | NA | low-risk |
| GSE84437 | GSM2236046 | 64 | NA | NA | NA | low-risk |
| GSE84437 | GSM2236047 | 49 | NA | NA | NA | low-risk |
| GSE84437 | GSM2236048 | 64 | NA | NA | NA | low-risk |
| GSE84437 | GSM2236049 | 44 | NA | NA | NA | low-risk |
| GSE84437 | GSM2236050 | 65 | NA | NA | NA | low-risk |
| GSE84437 | GSM2236051 | 34 | NA | NA | NA | high-risk |
| GSE84437 | GSM2236052 | 61 | NA | NA | NA | low-risk |
| GSE84437 | GSM2236053 | 41 | NA | NA | NA | high-risk |
| GSE84437 | GSM2236054 | 49 | NA | NA | NA | high-risk |
| GSE84437 | GSM2236055 | 55 | NA | NA | NA | low-risk |
| GSE84437 | GSM2236056 | 54 | NA | NA | NA | low-risk |
| GSE84437 | GSM2236057 | 62 | NA | NA | NA | low-risk |
| GSE84437 | GSM2236058 | 62 | NA | NA | NA | high-risk |
| GSE84437 | GSM2236059 | 75 | NA | NA | NA | high-risk |
| GSE84437 | GSM2236060 | 47 | NA | NA | NA | high-risk |
| GSE84437 | GSM2236061 | 68 | NA | NA | NA | low-risk |
| GSE84437 | GSM2236062 | 69 | NA | NA | NA | low-risk |
| GSE84437 | GSM2236063 | 48 | NA | NA | NA | low-risk |
| GSE84437 | GSM2236064 | 70 | NA | NA | NA | low-risk |
| GSE84437 | GSM2236065 | 58 | NA | NA | NA | high-risk |
| GSE84437 | GSM2236066 | 63 | NA | NA | NA | high-risk |
| GSE84437 | GSM2236067 | 47 | NA | NA | NA | low-risk |
| GSE84437 | GSM2236068 | 44 | NA | NA | NA | high-risk |
| GSE84437 | GSM2236069 | 69 | NA | NA | NA | low-risk |
| GSE84437 | GSM2236070 | 71 | NA | NA | NA | low-risk |
| GSE84437 | GSM2236071 | 54 | NA | NA | NA | low-risk |
| GSE84437 | GSM2236072 | 49 | NA | NA | NA | low-risk |
| GSE84437 | GSM2236073 | 60 | NA | NA | NA | high-risk |
| GSE84437 | GSM2236074 | 62 | NA | NA | NA | low-risk |
| GSE84437 | GSM2236075 | 44 | NA | NA | NA | high-risk |
| GSE84437 | GSM2236076 | 63 | NA | NA | NA | low-risk |
| GSE84437 | GSM2236077 | 68 | NA | NA | NA | low-risk |
| GSE84437 | GSM2236078 | 60 | NA | NA | NA | low-risk |
| GSE84437 | GSM2236079 | 67 | NA | NA | NA | low-risk |
| GSE84437 | GSM2236080 | 62 | NA | NA | NA | low-risk |
| GSE84437 | GSM2236082 | 53 | NA | NA | NA | low-risk |
| GSE84437 | GSM2236083 | 64 | NA | NA | NA | low-risk |
| GSE84437 | GSM2236084 | 59 | NA | NA | NA | low-risk |
| GSE84437 | GSM2236085 | 55 | NA | NA | NA | low-risk |
| GSE84437 | GSM2236086 | 36 | NA | NA | NA | low-risk |
| GSE84437 | GSM2236087 | 68 | NA | NA | NA | high-risk |
| GSE84437 | GSM2236088 | 61 | NA | NA | NA | high-risk |
| GSE84437 | GSM2236089 | 69 | NA | NA | NA | high-risk |
| GSE84437 | GSM2236090 | 86 | NA | NA | NA | high-risk |
| GSE84437 | GSM2236091 | 77 | NA | NA | NA | high-risk |
| GSE84437 | GSM2236092 | 58 | NA | NA | NA | high-risk |
| GSE84437 | GSM2236093 | 59 | NA | NA | NA | high-risk |
| GSE84437 | GSM2236094 | 64 | NA | NA | NA | high-risk |
| GSE84437 | GSM2236095 | 51 | NA | NA | NA | high-risk |
| GSE62254 | GSM1523727 | 67 | intestinal | 2 | MSI | low-risk |
| GSE62254 | GSM1523728 | 68 | intestinal | 2 | MSI | low-risk |
| GSE62254 | GSM1523729 | 42 | diffuse | 2 | TP53+ | low-risk |
| GSE62254 | GSM1523744 | 69 | diffuse | 2 | MSI | low-risk |
| GSE62254 | GSM1523745 | 68 | diffuse | 3 | TP53- | low-risk |
| GSE62254 | GSM1523746 | 56 | mixed | 2 | TP53- | low-risk |
| GSE62254 | GSM1523747 | 60 | mixed | 3 | TP53+ | low-risk |
| GSE62254 | GSM1523748 | 68 | diffuse | 3 | TP53+ | low-risk |
| GSE62254 | GSM1523765 | 52 | diffuse | 3 | TP53- | low-risk |
| GSE62254 | GSM1523768 | 52 | intestinal | 3 | TP53- | high-risk |
| GSE62254 | GSM1523769 | 71 | diffuse | 4 | TP53- | low-risk |
| GSE62254 | GSM1523770 | 63 | intestinal | 2 | MSI | high-risk |
| GSE62254 | GSM1523771 | 62 | diffuse | 4 | EMT | high-risk |
| GSE62254 | GSM1523772 | 58 | diffuse | 4 | EMT | high-risk |
| GSE62254 | GSM1523773 | 75 | diffuse | 4 | EMT | high-risk |
| GSE62254 | GSM1523774 | 56 | intestinal | 3 | EMT | low-risk |
| GSE62254 | GSM1523775 | 77 | diffuse | 2 | MSI | low-risk |
| GSE62254 | GSM1523776 | 48 | diffuse | 2 | MSI | low-risk |
| GSE62254 | GSM1523777 | 60 | intestinal | 4 | TP53- | high-risk |
| GSE62254 | GSM1523778 | 56 | diffuse | 3 | TP53- | low-risk |
| GSE62254 | GSM1523779 | 77 | diffuse | 3 | MSI | low-risk |
| GSE62254 | GSM1523780 | 84 | intestinal | 4 | MSI | high-risk |
| GSE62254 | GSM1523781 | 37 | intestinal | 4 | TP53- | low-risk |
| GSE62254 | GSM1523782 | 63 | intestinal | 4 | TP53+ | high-risk |
| GSE62254 | GSM1523783 | 55 | diffuse | 3 | TP53+ | low-risk |
| GSE62254 | GSM1523784 | 44 | diffuse | 3 | TP53- | low-risk |
| GSE62254 | GSM1523785 | 60 | intestinal | 2 | MSI | high-risk |
| GSE62254 | GSM1523786 | 44 | diffuse | 4 | TP53+ | low-risk |
| GSE62254 | GSM1523787 | 57 | intestinal | 4 | TP53+ | high-risk |
| GSE62254 | GSM1523788 | 42 | diffuse | 4 | EMT | low-risk |
| GSE62254 | GSM1523789 | 69 | diffuse | 4 | TP53- | high-risk |
| GSE62254 | GSM1523790 | 62 | intestinal | 2 | MSI | high-risk |
| GSE62254 | GSM1523791 | 60 | intestinal | 1 | MSI | high-risk |
| GSE62254 | GSM1523792 | 55 | intestinal | 1 | TP53- | high-risk |
| GSE62254 | GSM1523793 | 82 | diffuse | 2 | TP53+ | low-risk |
| GSE62254 | GSM1523794 | 67 | diffuse | 4 | MSI | high-risk |
| GSE62254 | GSM1523795 | 70 | intestinal | 1 | TP53+ | low-risk |
| GSE62254 | GSM1523796 | 53 | intestinal | 3 | EMT | high-risk |
| GSE62254 | GSM1523797 | 63 | intestinal | 3 | MSI | high-risk |
| GSE62254 | GSM1523798 | 84 | diffuse | 2 | EMT | low-risk |
| GSE62254 | GSM1523799 | 81 | intestinal | 2 | TP53+ | high-risk |
| GSE62254 | GSM1523800 | 73 | intestinal | 3 | MSI | low-risk |
| GSE62254 | GSM1523801 | 74 | indeterminate | 2 | MSI | low-risk |
| GSE62254 | GSM1523802 | 70 | intestinal | 2 | TP53- | low-risk |
| GSE62254 | GSM1523803 | 72 | indeterminate | 4 | TP53+ | low-risk |
| GSE62254 | GSM1523804 | 37 | diffuse | 3 | TP53+ | low-risk |
| GSE62254 | GSM1523805 | 72 | mixed | 3 | MSI | low-risk |
| GSE62254 | GSM1523806 | 68 | intestinal | 3 | TP53- | low-risk |
| GSE62254 | GSM1523807 | 70 | intestinal | 2 | TP53- | low-risk |
| GSE62254 | GSM1523808 | 56 | diffuse | 3 | MSI | low-risk |
| GSE62254 | GSM1523809 | 77 | intestinal | 2 | TP53+ | low-risk |
| GSE62254 | GSM1523810 | 66 | intestinal | 1 | MSI | high-risk |
| GSE62254 | GSM1523811 | 80 | intestinal | 1 | MSI | low-risk |
| GSE62254 | GSM1523812 | 62 | diffuse | 3 | TP53+ | low-risk |
| GSE62254 | GSM1523813 | 70 | diffuse | 3 | MSI | low-risk |
| GSE62254 | GSM1523814 | 68 | intestinal | 2 | TP53+ | low-risk |
| GSE62254 | GSM1523815 | 64 | mixed | 3 | TP53- | high-risk |
| GSE62254 | GSM1523816 | 66 | intestinal | 2 | TP53- | high-risk |
| GSE62254 | GSM1523817 | 73 | diffuse | 4 | TP53- | high-risk |
| GSE62254 | GSM1523818 | 66 | diffuse | 4 | TP53+ | high-risk |
| GSE62254 | GSM1523819 | 77 | intestinal | 4 | TP53+ | high-risk |
| GSE62254 | GSM1523820 | 64 | intestinal | 3 | TP53+ | low-risk |
| GSE62254 | GSM1523821 | 64 | intestinal | 4 | MSI | low-risk |
| GSE62254 | GSM1523822 | 66 | intestinal | 1 | MSI | low-risk |
| GSE62254 | GSM1523823 | 64 | intestinal | 3 | MSI | low-risk |
| GSE62254 | GSM1523824 | 64 | intestinal | 2 | TP53- | high-risk |
| GSE62254 | GSM1523825 | 69 | intestinal | 3 | MSI | high-risk |
| GSE62254 | GSM1523826 | 74 | intestinal | 3 | TP53- | low-risk |
| GSE62254 | GSM1523827 | 36 | diffuse | 3 | TP53- | low-risk |
| GSE62254 | GSM1523828 | 72 | intestinal | 4 | MSI | high-risk |
| GSE62254 | GSM1523829 | 68 | intestinal | 2 | TP53- | low-risk |
| GSE62254 | GSM1523830 | 59 | intestinal | 2 | MSI | low-risk |
| GSE62254 | GSM1523831 | 68 | diffuse | 4 | TP53+ | high-risk |
| GSE62254 | GSM1523832 | 62 | intestinal | 3 | TP53+ | low-risk |
| GSE62254 | GSM1523833 | 65 | diffuse | 4 | MSI | low-risk |
| GSE62254 | GSM1523834 | 66 | diffuse | 3 | TP53+ | high-risk |
| GSE62254 | GSM1523835 | 54 | intestinal | 2 | EMT | high-risk |
| GSE62254 | GSM1523836 | 56 | intestinal | 3 | TP53+ | low-risk |
| GSE62254 | GSM1523837 | 70 | intestinal | 1 | TP53- | low-risk |
| GSE62254 | GSM1523838 | 65 | intestinal | 3 | MSI | low-risk |
| GSE62254 | GSM1523839 | 57 | diffuse | 3 | TP53- | low-risk |
| GSE62254 | GSM1523840 | 78 | diffuse | 2 | TP53+ | low-risk |
| GSE62254 | GSM1523841 | 65 | mixed | 2 | TP53+ | high-risk |
| GSE62254 | GSM1523842 | 70 | diffuse | 2 | TP53- | low-risk |
| GSE62254 | GSM1523843 | 65 | diffuse | 4 | TP53- | low-risk |
| GSE62254 | GSM1523844 | 54 | intestinal | 3 | EMT | high-risk |
| GSE62254 | GSM1523845 | 77 | diffuse | 4 | TP53+ | high-risk |
| GSE62254 | GSM1523846 | 63 | diffuse | 3 | EMT | low-risk |
| GSE62254 | GSM1523847 | 31 | intestinal | 2 | MSI | high-risk |
| GSE62254 | GSM1523848 | 68 | diffuse | 3 | EMT | low-risk |
| GSE62254 | GSM1523849 | 65 | intestinal | 3 | EMT | high-risk |
| GSE62254 | GSM1523850 | 38 | diffuse | 4 | EMT | high-risk |
| GSE62254 | GSM1523851 | 77 | intestinal | 1 | MSI | low-risk |
| GSE62254 | GSM1523852 | 55 | intestinal | 3 | TP53- | low-risk |
| GSE62254 | GSM1523853 | 68 | intestinal | 1 | TP53- | low-risk |
| GSE62254 | GSM1523854 | 69 | diffuse | 2 | TP53- | low-risk |
| GSE62254 | GSM1523855 | 77 | intestinal | 3 | TP53- | high-risk |
| GSE62254 | GSM1523856 | 51 | diffuse | 2 | MSI | high-risk |
| GSE62254 | GSM1523857 | 75 | intestinal | 3 | TP53- | high-risk |
| GSE62254 | GSM1523858 | 42 | diffuse | 1 | EMT | high-risk |
| GSE62254 | GSM1523859 | 67 | intestinal | 2 | TP53+ | low-risk |
| GSE62254 | GSM1523860 | 66 | mixed | 1 | MSI | high-risk |
| GSE62254 | GSM1523861 | 64 | intestinal | 1 | TP53+ | low-risk |
| GSE62254 | GSM1523862 | 41 | diffuse | 4 | EMT | high-risk |
| GSE62254 | GSM1523863 | 64 | mixed | 1 | MSI | low-risk |
| GSE62254 | GSM1523864 | 52 | intestinal | 1 | MSI | low-risk |
| GSE62254 | GSM1523865 | 70 | intestinal | 2 | TP53- | high-risk |
| GSE62254 | GSM1523866 | 86 | intestinal | 4 | EMT | low-risk |
| GSE62254 | GSM1523867 | 54 | intestinal | 3 | MSI | low-risk |
| GSE62254 | GSM1523868 | 62 | mixed | 3 | MSI | high-risk |
| GSE62254 | GSM1523869 | 67 | diffuse | 2 | TP53+ | low-risk |
| GSE62254 | GSM1523870 | 75 | intestinal | 2 | MSI | high-risk |
| GSE62254 | GSM1523871 | 70 | intestinal | 2 | MSI | low-risk |
| GSE62254 | GSM1523872 | 61 | diffuse | 4 | TP53- | low-risk |
| GSE62254 | GSM1523873 | 57 | diffuse | 4 | TP53+ | high-risk |
| GSE62254 | GSM1523874 | 72 | intestinal | 1 | TP53- | high-risk |
| GSE62254 | GSM1523875 | 79 | intestinal | 2 | TP53+ | high-risk |
| GSE62254 | GSM1523876 | 76 | intestinal | 1 | MSI | low-risk |
| GSE62254 | GSM1523877 | 65 | intestinal | 3 | TP53- | high-risk |
| GSE62254 | GSM1523878 | 56 | intestinal | 2 | TP53+ | low-risk |
| GSE62254 | GSM1523879 | 63 | intestinal | 4 | TP53- | low-risk |
| GSE62254 | GSM1523880 | 54 | diffuse | 1 | MSI | low-risk |
| GSE62254 | GSM1523881 | 74 | intestinal | 3 | TP53+ | low-risk |
| GSE62254 | GSM1523882 | 36 | diffuse | 3 | TP53+ | low-risk |
| GSE62254 | GSM1523883 | 77 | diffuse | 2 | TP53+ | low-risk |
| GSE62254 | GSM1523884 | 50 | diffuse | 3 | TP53+ | low-risk |
| GSE62254 | GSM1523885 | 53 | diffuse | 1 | MSI | low-risk |
| GSE62254 | GSM1523886 | 49 | diffuse | 4 | EMT | high-risk |
| GSE62254 | GSM1523887 | 46 | diffuse | 1 | TP53- | low-risk |
| GSE62254 | GSM1523888 | 52 | intestinal | 1 | MSI | low-risk |
| GSE62254 | GSM1523889 | 74 | intestinal | 1 | MSI | high-risk |
| GSE62254 | GSM1523890 | 33 | diffuse | 3 | TP53- | low-risk |
| GSE62254 | GSM1523891 | 53 | intestinal | 1 | TP53- | low-risk |
| GSE62254 | GSM1523892 | 59 | intestinal | 1 | TP53- | low-risk |
| GSE62254 | GSM1523893 | 74 | intestinal | 1 | TP53- | high-risk |
| GSE62254 | GSM1523894 | 56 | intestinal | 1 | TP53+ | high-risk |
| GSE62254 | GSM1523895 | 66 | intestinal | 1 | TP53- | high-risk |
| GSE62254 | GSM1523896 | 51 | intestinal | 2 | TP53+ | low-risk |
| GSE62254 | GSM1523897 | 78 | intestinal | 1 | TP53- | high-risk |
| GSE62254 | GSM1523898 | 60 | intestinal | 1 | TP53+ | low-risk |
| GSE62254 | GSM1523899 | 60 | intestinal | 2 | TP53+ | high-risk |
| GSE62254 | GSM1523901 | 65 | intestinal | 1 | MSI | low-risk |
| GSE62254 | GSM1523903 | 60 | intestinal | 3 | MSI | high-risk |
| GSE62254 | GSM1523904 | 56 | intestinal | 4 | TP53- | high-risk |
| GSE62254 | GSM1523905 | 66 | diffuse | 2 | TP53- | low-risk |
| GSE62254 | GSM1523906 | 53 | diffuse | 1 | EMT | low-risk |
| GSE62254 | GSM1523908 | 44 | diffuse | 4 | TP53- | high-risk |
| GSE62254 | GSM1523909 | 74 | diffuse | 2 | MSI | low-risk |
| GSE62254 | GSM1523910 | 53 | diffuse | 3 | MSI | low-risk |
| GSE62254 | GSM1523911 | 72 | intestinal | 4 | MSI | high-risk |
| GSE62254 | GSM1523918 | 62 | diffuse | 3 | EMT | high-risk |
| GSE62254 | GSM1523919 | 64 | intestinal | 4 | TP53+ | high-risk |
| GSE62254 | GSM1523920 | 44 | intestinal | 2 | TP53- | low-risk |
| GSE62254 | GSM1523921 | 42 | diffuse | 2 | MSI | high-risk |
| GSE62254 | GSM1523922 | 65 | intestinal | 2 | TP53- | high-risk |
| GSE62254 | GSM1523923 | 59 | diffuse | 2 | TP53+ | low-risk |
| GSE62254 | GSM1523924 | 52 | diffuse | 2 | TP53- | low-risk |
| GSE62254 | GSM1523925 | 59 | diffuse | 2 | TP53- | low-risk |
| GSE62254 | GSM1523926 | 69 | intestinal | 1 | TP53+ | high-risk |
| GSE62254 | GSM1523927 | 46 | diffuse | 2 | TP53+ | low-risk |
| GSE62254 | GSM1523928 | 69 | intestinal | 2 | TP53- | high-risk |
| GSE62254 | GSM1523929 | 67 | intestinal | 3 | MSI | low-risk |
| GSE62254 | GSM1523930 | 51 | intestinal | 2 | TP53- | low-risk |
| GSE62254 | GSM1523931 | 61 | diffuse | 2 | TP53+ | low-risk |
| GSE62254 | GSM1523932 | 66 | intestinal | 2 | TP53- | low-risk |
| GSE62254 | GSM1523933 | 74 | diffuse | 2 | MSI | high-risk |
| GSE62254 | GSM1523934 | 70 | intestinal | 4 | TP53- | high-risk |
| GSE62254 | GSM1523935 | 64 | diffuse | 2 | EMT | high-risk |
| GSE62254 | GSM1523936 | 70 | diffuse | 2 | TP53+ | high-risk |
| GSE62254 | GSM1523937 | 81 | intestinal | 2 | TP53+ | low-risk |
| GSE62254 | GSM1523938 | 53 | diffuse | 4 | EMT | high-risk |
| GSE62254 | GSM1523939 | 28 | diffuse | 4 | EMT | high-risk |
| GSE62254 | GSM1523940 | 69 | intestinal | 4 | TP53- | high-risk |
| GSE62254 | GSM1523941 | 70 | diffuse | 4 | TP53- | high-risk |
| GSE62254 | GSM1523942 | 70 | intestinal | 2 | TP53- | low-risk |
| GSE62254 | GSM1523943 | 72 | diffuse | 4 | TP53- | low-risk |
| GSE62254 | GSM1523944 | 71 | diffuse | 3 | TP53+ | low-risk |
| GSE62254 | GSM1523945 | 53 | diffuse | 4 | EMT | high-risk |
| GSE62254 | GSM1523946 | 50 | intestinal | 3 | TP53+ | high-risk |
| GSE62254 | GSM1523947 | 74 | intestinal | 2 | MSI | low-risk |
| GSE62254 | GSM1523948 | 52 | diffuse | 4 | EMT | high-risk |
| GSE62254 | GSM1523949 | 51 | diffuse | 4 | EMT | high-risk |
| GSE62254 | GSM1523950 | 57 | intestinal | 4 | TP53- | low-risk |
| GSE62254 | GSM1523951 | 66 | diffuse | 4 | TP53+ | low-risk |
| GSE62254 | GSM1523952 | 54 | diffuse | 2 | MSI | low-risk |
| GSE62254 | GSM1523953 | 70 | diffuse | 3 | TP53- | high-risk |
| GSE62254 | GSM1523954 | 62 | diffuse | 2 | TP53- | low-risk |
| GSE62254 | GSM1523955 | 49 | mixed | 3 | TP53+ | low-risk |
| GSE62254 | GSM1523956 | 76 | mixed | 2 | TP53- | high-risk |
| GSE62254 | GSM1523957 | 37 | intestinal | 2 | TP53+ | high-risk |
| GSE62254 | GSM1523958 | 56 | diffuse | 2 | TP53+ | low-risk |
| GSE62254 | GSM1523959 | 54 | intestinal | 4 | TP53- | high-risk |
| GSE62254 | GSM1523960 | 70 | intestinal | 2 | TP53- | high-risk |
| GSE62254 | GSM1523961 | 63 | intestinal | 2 | MSI | high-risk |
| GSE62254 | GSM1523962 | 48 | diffuse | 3 | EMT | high-risk |
| GSE62254 | GSM1523963 | 69 | intestinal | 2 | TP53- | low-risk |
| GSE62254 | GSM1523964 | 61 | intestinal | 2 | MSI | low-risk |
| GSE62254 | GSM1523965 | 52 | diffuse | 4 | EMT | high-risk |
| GSE62254 | GSM1523966 | 35 | diffuse | 4 | TP53- | low-risk |
| GSE62254 | GSM1523967 | 52 | diffuse | 3 | TP53+ | low-risk |
| GSE62254 | GSM1523968 | 44 | diffuse | 4 | EMT | high-risk |
| GSE62254 | GSM1523969 | 69 | diffuse | 3 | TP53- | high-risk |
| GSE62254 | GSM1523970 | 77 | mixed | 2 | TP53- | high-risk |
| GSE62254 | GSM1523971 | 45 | diffuse | 3 | EMT | low-risk |
| GSE62254 | GSM1523972 | 70 | mixed | 4 | TP53- | high-risk |
| GSE62254 | GSM1523973 | 62 | diffuse | 3 | TP53- | high-risk |
| GSE62254 | GSM1523974 | 61 | intestinal | 2 | TP53- | low-risk |
| GSE62254 | GSM1523975 | 67 | diffuse | 3 | EMT | high-risk |
| GSE62254 | GSM1523976 | 69 | mixed | 3 | TP53- | low-risk |
| GSE62254 | GSM1523977 | 63 | diffuse | 2 | TP53- | high-risk |
| GSE62254 | GSM1523978 | 67 | diffuse | 4 | TP53+ | low-risk |
| GSE62254 | GSM1523979 | 78 | intestinal | 3 | MSI | low-risk |
| GSE62254 | GSM1523980 | 77 | diffuse | 3 | TP53+ | low-risk |
| GSE62254 | GSM1523981 | 51 | diffuse | 2 | EMT | high-risk |
| GSE62254 | GSM1523982 | 56 | diffuse | 4 | EMT | high-risk |
| GSE62254 | GSM1523983 | 61 | diffuse | 3 | MSI | high-risk |
| GSE62254 | GSM1523984 | 57 | diffuse | 2 | EMT | high-risk |
| GSE62254 | GSM1523985 | 70 | intestinal | 3 | TP53+ | low-risk |
| GSE62254 | GSM1523986 | 72 | diffuse | 3 | TP53+ | low-risk |
| GSE62254 | GSM1523987 | 30 | diffuse | 3 | TP53- | low-risk |
| GSE62254 | GSM1523988 | 65 | diffuse | 3 | EMT | high-risk |
| GSE62254 | GSM1523989 | 46 | intestinal | 4 | TP53- | high-risk |
| GSE62254 | GSM1523990 | 40 | diffuse | 3 | EMT | high-risk |
| GSE62254 | GSM1523991 | 63 | intestinal | 2 | TP53- | high-risk |
| GSE62254 | GSM1523992 | 70 | diffuse | 4 | TP53+ | low-risk |
| GSE62254 | GSM1523993 | 69 | intestinal | 3 | TP53- | low-risk |
| GSE62254 | GSM1523994 | 63 | intestinal | 3 | TP53- | low-risk |
| GSE62254 | GSM1523995 | 82 | mixed | 3 | TP53- | high-risk |
| GSE62254 | GSM1523996 | 75 | diffuse | 2 | TP53+ | low-risk |
| GSE62254 | GSM1523997 | 72 | diffuse | 4 | TP53- | high-risk |
| GSE62254 | GSM1523998 | 59 | intestinal | 3 | TP53- | high-risk |
| GSE62254 | GSM1523999 | 66 | intestinal | 2 | TP53- | low-risk |
| GSE62254 | GSM1524000 | 69 | diffuse | 3 | MSI | low-risk |
| GSE62254 | GSM1524001 | 58 | intestinal | 2 | TP53- | low-risk |
| GSE62254 | GSM1524002 | 64 | mixed | 3 | MSI | low-risk |
| GSE62254 | GSM1524003 | 60 | intestinal | 3 | TP53- | low-risk |
| GSE62254 | GSM1524004 | 58 | diffuse | 4 | TP53- | low-risk |
| GSE62254 | GSM1524005 | 71 | intestinal | 4 | TP53- | low-risk |
| GSE62254 | GSM1524006 | 41 | diffuse | 3 | EMT | high-risk |
| GSE62254 | GSM1524007 | 48 | diffuse | 4 | TP53- | low-risk |
| GSE62254 | GSM1524008 | 39 | diffuse | 4 | MSI | high-risk |
| GSE62254 | GSM1524009 | 60 | diffuse | 3 | TP53- | low-risk |
| GSE62254 | GSM1524010 | 73 | diffuse | 3 | EMT | high-risk |
| GSE62254 | GSM1524011 | 63 | intestinal | 2 | TP53- | high-risk |
| GSE62254 | GSM1524012 | 64 | diffuse | 3 | TP53- | high-risk |
| GSE62254 | GSM1524013 | 63 | intestinal | 2 | TP53- | low-risk |
| GSE62254 | GSM1524014 | 66 | intestinal | 4 | TP53- | high-risk |
| GSE62254 | GSM1524015 | 61 | intestinal | 4 | TP53- | high-risk |
| GSE62254 | GSM1524016 | 24 | diffuse | 4 | TP53+ | low-risk |
| GSE62254 | GSM1524017 | 67 | intestinal | 3 | TP53- | low-risk |
| GSE62254 | GSM1524018 | 64 | mixed | 3 | TP53+ | low-risk |
| GSE62254 | GSM1524019 | 70 | diffuse | 4 | TP53- | high-risk |
| GSE62254 | GSM1524020 | 51 | intestinal | 3 | TP53+ | high-risk |
| GSE62254 | GSM1524021 | 56 | diffuse | 3 | TP53+ | low-risk |
| GSE62254 | GSM1524022 | 65 | diffuse | 4 | TP53- | high-risk |
| GSE62254 | GSM1524023 | 77 | intestinal | 4 | TP53- | high-risk |
| GSE62254 | GSM1524024 | 61 | diffuse | 3 | TP53+ | low-risk |
| GSE62254 | GSM1524025 | 71 | diffuse | 3 | MSI | high-risk |
| GSE62254 | GSM1524026 | 52 | diffuse | 3 | EMT | high-risk |
| GSE62254 | GSM1524027 | 61 | diffuse | 3 | TP53- | high-risk |
| GSE62254 | GSM1524028 | 72 | intestinal | 3 | TP53+ | high-risk |
| GSE62254 | GSM1524029 | 50 | diffuse | 4 | EMT | high-risk |
| GSE62254 | GSM1524030 | 46 | diffuse | 3 | EMT | high-risk |
| GSE62254 | GSM1524031 | 55 | intestinal | 4 | TP53+ | high-risk |
| GSE62254 | GSM1524032 | 70 | intestinal | 4 | MSI | high-risk |
| GSE62254 | GSM1524033 | 67 | intestinal | 4 | TP53+ | low-risk |
| GSE62254 | GSM1524034 | 65 | intestinal | 3 | TP53+ | low-risk |
| GSE62254 | GSM1524035 | 64 | mixed | 4 | EMT | high-risk |
| GSE62254 | GSM1524036 | 70 | intestinal | 3 | TP53- | low-risk |
| GSE62254 | GSM1524037 | 72 | intestinal | 3 | TP53- | high-risk |
| GSE62254 | GSM1524038 | 74 | diffuse | 4 | TP53+ | high-risk |
| GSE62254 | GSM1524039 | 72 | diffuse | 3 | EMT | high-risk |
| GSE62254 | GSM1524040 | 55 | diffuse | 4 | TP53- | high-risk |
| GSE62254 | GSM1524041 | 56 | intestinal | 4 | MSI | high-risk |
| GSE62254 | GSM1524042 | 62 | diffuse | 3 | TP53- | low-risk |
| GSE62254 | GSM1524043 | 60 | diffuse | 4 | TP53+ | high-risk |
| GSE62254 | GSM1524044 | 80 | intestinal | 4 | TP53- | high-risk |
| GSE62254 | GSM1524045 | 33 | intestinal | 2 | TP53+ | low-risk |
| GSE62254 | GSM1524046 | 59 | diffuse | 2 | TP53- | high-risk |
| GSE62254 | GSM1524047 | 74 | diffuse | 2 | TP53+ | high-risk |
| GSE62254 | GSM1524048 | 67 | intestinal | 2 | MSI | high-risk |
| GSE62254 | GSM1524049 | 62 | intestinal | 2 | TP53+ | high-risk |
| GSE62254 | GSM1524050 | 66 | intestinal | 2 | TP53+ | low-risk |
| GSE62254 | GSM1524051 | 51 | diffuse | 2 | TP53- | low-risk |
| GSE62254 | GSM1524052 | 74 | diffuse | 2 | TP53- | high-risk |
| GSE62254 | GSM1524053 | 61 | intestinal | 2 | TP53+ | high-risk |
| GSE62254 | GSM1524054 | 72 | intestinal | 2 | MSI | high-risk |
| GSE62254 | GSM1524055 | 54 | intestinal | 2 | TP53+ | high-risk |
| GSE62254 | GSM1524056 | 71 | intestinal | 2 | TP53- | high-risk |
| GSE62254 | GSM1524057 | 80 | intestinal | 4 | EMT | high-risk |
| GSE62254 | GSM1524058 | 56 | intestinal | 2 | EMT | high-risk |
| GSE62254 | GSM1524059 | 58 | diffuse | 2 | EMT | high-risk |
| GSE62254 | GSM1524060 | 56 | intestinal | 2 | TP53+ | high-risk |
| GSE62254 | GSM1524061 | 77 | intestinal | 2 | MSI | high-risk |
| GSE62254 | GSM1524062 | 40 | diffuse | 3 | EMT | high-risk |
| GSE62254 | GSM1524068 | 71 | intestinal | 2 | MSI | high-risk |
| GSE62254 | GSM1524069 | 63 | intestinal | 2 | MSI | low-risk |
| GSE62254 | GSM1524070 | 58 | intestinal | 2 | TP53+ | high-risk |
| GSE62254 | GSM1524071 | 60 | intestinal | 3 | TP53+ | high-risk |
| GSE62254 | GSM1524072 | 43 | diffuse | 4 | TP53- | high-risk |
| GSE26901 | GSM662576 | 28 | intestinal | 4 | NA | low-risk |
| GSE26901 | GSM662564 | 71 | intestinal | 1 | NA | high-risk |
| GSE26901 | GSM662554 | 66 | mixed | 2 | NA | low-risk |
| GSE26901 | GSM662527 | 31 | intestinal | 3 | NA | low-risk |
| GSE26901 | GSM662528 | 63 | intestinal | 4 | NA | high-risk |
| GSE26901 | GSM662565 | 49 | intestinal | 3 | NA | low-risk |
| GSE26901 | GSM662566 | 67 | NA | 1 | NA | high-risk |
| GSE26901 | GSM662555 | 67 | intestinal | 3 | NA | low-risk |
| GSE26901 | GSM662577 | 63 | intestinal | 4 | NA | high-risk |
| GSE26901 | GSM662556 | 58 | intestinal | 2 | NA | low-risk |
| GSE26901 | GSM662545 | 45 | intestinal | 1 | NA | high-risk |
| GSE26901 | GSM662578 | 55 | intestinal | 2 | NA | low-risk |
| GSE26901 | GSM662546 | 35 | intestinal | 3 | NA | low-risk |
| GSE26901 | GSM662557 | 68 | intestinal | 2 | NA | high-risk |
| GSE26901 | GSM662547 | 64 | intestinal | 4 | NA | low-risk |
| GSE26901 | GSM662579 | 54 | intestinal | 3 | NA | high-risk |
| GSE26901 | GSM662590 | 65 | intestinal | 4 | NA | high-risk |
| GSE26901 | GSM662591 | 50 | intestinal | 1 | NA | high-risk |
| GSE26901 | GSM662580 | 68 | intestinal | 3 | NA | high-risk |
| GSE26901 | GSM662529 | 54 | intestinal | 4 | NA | high-risk |
| GSE26901 | GSM662543 | 60 | intestinal | 1 | NA | high-risk |
| GSE26901 | GSM662544 | 58 | intestinal | 1 | NA | low-risk |
| GSE26901 | GSM662530 | 58 | intestinal | 1 | NA | high-risk |
| GSE26901 | GSM662567 | 58 | intestinal | 1 | NA | high-risk |
| GSE26901 | GSM662509 | 69 | intestinal | 1 | NA | low-risk |
| GSE26901 | GSM662531 | 67 | intestinal | 3 | NA | high-risk |
| GSE26901 | GSM662549 | 45 | diffuse | 1 | NA | high-risk |
| GSE26901 | GSM662550 | 68 | intestinal | 4 | NA | high-risk |
| GSE26901 | GSM662596 | 38 | diffuse | 2 | NA | high-risk |
| GSE26901 | GSM662551 | 63 | intestinal | 2 | NA | high-risk |
| GSE26901 | GSM662581 | 65 | intestinal | 3 | NA | high-risk |
| GSE26901 | GSM662552 | 49 | intestinal | 3 | NA | high-risk |
| GSE26901 | GSM662582 | 49 | NA | 4 | NA | high-risk |
| GSE26901 | GSM662532 | 68 | NA | 1 | NA | high-risk |
| GSE26901 | GSM662583 | 64 | intestinal | 1 | NA | high-risk |
| GSE26901 | GSM662568 | 49 | intestinal | 4 | NA | low-risk |
| GSE26901 | GSM662533 | 51 | intestinal | 1 | NA | high-risk |
| GSE26901 | GSM662597 | 64 | intestinal | 1 | NA | low-risk |
| GSE26901 | GSM662584 | 68 | intestinal | 1 | NA | high-risk |
| GSE26901 | GSM662592 | 48 | intestinal | 3 | NA | high-risk |
| GSE26901 | GSM662585 | 60 | intestinal | 1 | NA | high-risk |
| GSE26901 | GSM662534 | 53 | intestinal | 3 | NA | high-risk |
| GSE26901 | GSM662511 | 59 | diffuse | 1 | NA | low-risk |
| GSE26901 | GSM662598 | 70 | intestinal | 3 | NA | low-risk |
| GSE26901 | GSM662599 | 47 | diffuse | 3 | NA | high-risk |
| GSE26901 | GSM662586 | 74 | intestinal | 3 | NA | high-risk |
| GSE26901 | GSM662594 | 47 | NA | 1 | NA | low-risk |
| GSE26901 | GSM662600 | 59 | NA | 1 | NA | high-risk |
| GSE26901 | GSM662601 | 42 | intestinal | 3 | NA | high-risk |
| GSE26901 | GSM662587 | 53 | diffuse | 3 | NA | high-risk |
| GSE26901 | GSM662570 | 46 | intestinal | 4 | NA | low-risk |
| GSE26901 | GSM662558 | 55 | intestinal | 1 | NA | low-risk |
| GSE26901 | GSM662595 | 43 | intestinal | 2 | NA | low-risk |
| GSE26901 | GSM662514 | 30 | diffuse | 3 | NA | low-risk |
| GSE26901 | GSM662515 | 66 | mixed | 3 | NA | low-risk |
| GSE26901 | GSM662516 | 34 | NA | 2 | NA | high-risk |
| GSE26901 | GSM662571 | 52 | intestinal | 3 | NA | low-risk |
| GSE26901 | GSM662536 | 50 | NA | 1 | NA | low-risk |
| GSE26901 | GSM662572 | 71 | intestinal | 3 | NA | high-risk |
| GSE26901 | GSM662559 | 67 | intestinal | 3 | NA | high-risk |
| GSE26901 | GSM662573 | 69 | intestinal | 2 | NA | low-risk |
| GSE26901 | GSM662560 | 52 | intestinal | 2 | NA | low-risk |
| GSE26901 | GSM662561 | 72 | intestinal | 1 | NA | low-risk |
| GSE26901 | GSM662518 | 67 | diffuse | 1 | NA | low-risk |
| GSE26901 | GSM662602 | 73 | intestinal | 3 | NA | high-risk |
| GSE26901 | GSM662562 | 55 | intestinal | 2 | NA | low-risk |
| GSE26901 | GSM662574 | 65 | intestinal | 4 | NA | high-risk |
| GSE26901 | GSM662519 | 54 | intestinal | 2 | NA | low-risk |
| GSE26901 | GSM662575 | 48 | intestinal | 3 | NA | high-risk |
| GSE26901 | GSM662537 | 61 | intestinal | 1 | NA | low-risk |
| GSE26901 | GSM662563 | 59 | intestinal | 1 | NA | low-risk |
| GSE26901 | GSM662520 | 47 | intestinal | 2 | NA | low-risk |
| GSE26901 | GSM662612 | 67 | mixed | 4 | NA | high-risk |
| GSE26901 | GSM662539 | 51 | intestinal | 3 | NA | low-risk |
| GSE26901 | GSM662540 | 63 | intestinal | 4 | NA | low-risk |
| GSE26901 | GSM662603 | 66 | intestinal | 2 | NA | high-risk |
| GSE26901 | GSM662521 | 58 | mixed | 4 | NA | low-risk |
| GSE26901 | GSM662522 | 50 | NA | 1 | NA | low-risk |
| GSE26901 | GSM662588 | 59 | intestinal | 1 | NA | high-risk |
| GSE26901 | GSM662523 | 64 | intestinal | 1 | NA | low-risk |
| GSE26901 | GSM662604 | 63 | intestinal | 1 | NA | low-risk |
| GSE26901 | GSM662613 | 29 | intestinal | 1 | NA | high-risk |
| GSE26901 | GSM662614 | 55 | NA | 3 | NA | low-risk |
| GSE26901 | GSM662524 | 70 | intestinal | 4 | NA | low-risk |
| GSE26901 | GSM662605 | 60 | intestinal | 3 | NA | low-risk |
| GSE26901 | GSM662606 | 61 | intestinal | 3 | NA | low-risk |
| GSE26901 | GSM662589 | 59 | intestinal | 1 | NA | low-risk |
| GSE26901 | GSM662607 | 41 | NA | 2 | NA | high-risk |
| GSE26901 | GSM662525 | 58 | intestinal | 3 | NA | high-risk |
| GSE26901 | GSM662609 | 53 | diffuse | 2 | NA | low-risk |
| GSE26901 | GSM662615 | 55 | mixed | 3 | NA | low-risk |
| GSE26901 | GSM662610 | 50 | diffuse | 1 | NA | high-risk |
| GSE26901 | GSM662616 | 67 | intestinal | 3 | NA | high-risk |
| GSE26901 | GSM662617 | 47 | intestinal | 1 | NA | high-risk |
| GSE26901 | GSM662611 | 62 | NA | 3 | NA | low-risk |
| GSE26901 | GSM662541 | 52 | diffuse | 3 | NA | low-risk |
| GSE26901 | GSM662526 | 40 | intestinal | 2 | NA | low-risk |
| GSE29272 | GSM723609 | 52 | NA | 3 | NA | high-risk |
| GSE29272 | GSM723611 | 37 | NA | 3 | NA | high-risk |
| GSE29272 | GSM723613 | 61 | NA | 3 | NA | high-risk |
| GSE29272 | GSM723615 | 57 | NA | 3 | NA | high-risk |
| GSE29272 | GSM723617 | 69 | NA | 3 | NA | low-risk |
| GSE29272 | GSM723619 | 71 | NA | 3 | NA | low-risk |
| GSE29272 | GSM723621 | 73 | NA | 3 | NA | low-risk |
| GSE29272 | GSM723623 | 37 | NA | 3 | NA | low-risk |
| GSE29272 | GSM723625 | 62 | NA | 3 | NA | low-risk |
| GSE29272 | GSM723627 | 66 | NA | 3 | NA | low-risk |
| GSE29272 | GSM723629 | 61 | NA | 3 | NA | low-risk |
| GSE29272 | GSM723631 | 35 | NA | 3 | NA | low-risk |
| GSE29272 | GSM723633 | 57 | NA | 3 | NA | low-risk |
| GSE29272 | GSM723635 | 60 | NA | 4 | NA | high-risk |
| GSE29272 | GSM723637 | 58 | NA | 3 | NA | low-risk |
| GSE29272 | GSM723639 | 58 | NA | 3 | NA | low-risk |
| GSE29272 | GSM723641 | 62 | NA | 3 | NA | high-risk |
| GSE29272 | GSM723643 | 59 | NA | 3 | NA | low-risk |
| GSE29272 | GSM723645 | 69 | NA | 3 | NA | low-risk |
| GSE29272 | GSM723647 | 68 | NA | 3 | NA | low-risk |
| GSE29272 | GSM723649 | 67 | NA | 3 | NA | high-risk |
| GSE29272 | GSM723651 | 61 | NA | 3 | NA | low-risk |
| GSE29272 | GSM723653 | 62 | NA | 3 | NA | low-risk |
| GSE29272 | GSM723655 | 69 | NA | 3 | NA | high-risk |
| GSE29272 | GSM723657 | 60 | NA | 3 | NA | low-risk |
| GSE29272 | GSM723659 | 58 | NA | 3 | NA | low-risk |
| GSE29272 | GSM723661 | 58 | NA | 3 | NA | low-risk |
| GSE29272 | GSM723663 | 35 | NA | 3 | NA | low-risk |
| GSE29272 | GSM723665 | 62 | NA | 3 | NA | low-risk |
| GSE29272 | GSM723667 | 66 | NA | 3 | NA | low-risk |
| GSE29272 | GSM723669 | 65 | NA | 3 | NA | high-risk |
| GSE29272 | GSM723671 | 67 | NA | 3 | NA | high-risk |
| GSE29272 | GSM723673 | 67 | NA | 3 | NA | high-risk |
| GSE29272 | GSM723675 | 48 | NA | 1 | NA | low-risk |
| GSE29272 | GSM723677 | 52 | NA | 3 | NA | low-risk |
| GSE29272 | GSM723679 | 53 | NA | 3 | NA | low-risk |
| GSE29272 | GSM723681 | 57 | NA | 4 | NA | high-risk |
| GSE29272 | GSM723683 | 59 | NA | 3 | NA | low-risk |
| GSE29272 | GSM723685 | 68 | NA | 4 | NA | high-risk |
| GSE29272 | GSM723687 | 46 | NA | 3 | NA | high-risk |
| GSE29272 | GSM723689 | 64 | NA | 3 | NA | low-risk |
| GSE29272 | GSM723691 | 55 | NA | 4 | NA | high-risk |
| GSE29272 | GSM723693 | 62 | NA | 3 | NA | high-risk |
| GSE29272 | GSM723695 | 66 | NA | 3 | NA | high-risk |
| GSE29272 | GSM723697 | 69 | NA | 3 | NA | high-risk |
| GSE29272 | GSM723699 | 23 | NA | 3 | NA | low-risk |
| GSE29272 | GSM723701 | 59 | NA | 3 | NA | low-risk |
| GSE29272 | GSM723703 | 62 | NA | 3 | NA | high-risk |
| GSE29272 | GSM723705 | 54 | NA | 3 | NA | high-risk |
| GSE29272 | GSM723707 | 41 | NA | 3 | NA | low-risk |
| GSE29272 | GSM723709 | 65 | NA | 3 | NA | low-risk |
| GSE29272 | GSM723711 | 58 | NA | 3 | NA | low-risk |
| GSE29272 | GSM723713 | 68 | NA | 3 | NA | low-risk |
| GSE29272 | GSM723715 | 61 | NA | 3 | NA | low-risk |
| GSE29272 | GSM723717 | 65 | NA | 3 | NA | low-risk |
| GSE29272 | GSM723719 | 51 | NA | 3 | NA | low-risk |
| GSE29272 | GSM723721 | 63 | NA | 3 | NA | high-risk |
| GSE29272 | GSM723723 | 66 | NA | 3 | NA | high-risk |
| GSE29272 | GSM723725 | 68 | NA | 3 | NA | low-risk |
| GSE29272 | GSM723727 | 55 | NA | 3 | NA | high-risk |
| GSE29272 | GSM723729 | 59 | NA | 4 | NA | low-risk |
| GSE29272 | GSM723731 | 50 | NA | 3 | NA | high-risk |
| GSE29272 | GSM723465 | 56 | NA | 3 | NA | high-risk |
| GSE29272 | GSM723467 | 49 | NA | 3 | NA | high-risk |
| GSE29272 | GSM723469 | 57 | NA | 3 | NA | high-risk |
| GSE29272 | GSM723471 | 65 | NA | 3 | NA | high-risk |
| GSE29272 | GSM723473 | 54 | NA | 3 | NA | high-risk |
| GSE29272 | GSM723475 | 61 | NA | 3 | NA | high-risk |
| GSE29272 | GSM723477 | 55 | NA | 3 | NA | high-risk |
| GSE29272 | GSM723479 | 66 | NA | 3 | NA | high-risk |
| GSE29272 | GSM723481 | 59 | NA | 3 | NA | high-risk |
| GSE29272 | GSM723483 | 60 | NA | 3 | NA | high-risk |
| GSE29272 | GSM723485 | 52 | NA | 3 | NA | low-risk |
| GSE29272 | GSM723487 | 62 | NA | 3 | NA | high-risk |
| GSE29272 | GSM723489 | 70 | NA | 3 | NA | low-risk |
| GSE29272 | GSM723491 | 64 | NA | 3 | NA | high-risk |
| GSE29272 | GSM723493 | 35 | NA | 3 | NA | high-risk |
| GSE29272 | GSM723495 | 58 | NA | 4 | NA | high-risk |
| GSE29272 | GSM723497 | 64 | NA | 3 | NA | low-risk |
| GSE29272 | GSM723499 | 63 | NA | 3 | NA | high-risk |
| GSE29272 | GSM723501 | 59 | NA | 3 | NA | low-risk |
| GSE29272 | GSM723503 | 27 | NA | 4 | NA | high-risk |
| GSE29272 | GSM723505 | 42 | NA | 3 | NA | low-risk |
| GSE29272 | GSM723507 | 28 | NA | 3 | NA | high-risk |
| GSE29272 | GSM723509 | 59 | NA | 4 | NA | high-risk |
| GSE29272 | GSM723511 | 65 | NA | 3 | NA | low-risk |
| GSE29272 | GSM723513 | 28 | NA | 3 | NA | high-risk |
| GSE29272 | GSM723515 | 38 | NA | 3 | NA | low-risk |
| GSE29272 | GSM723517 | 61 | NA | 2 | NA | low-risk |
| GSE29272 | GSM723519 | 57 | NA | 3 | NA | low-risk |
| GSE29272 | GSM723521 | 58 | NA | 3 | NA | high-risk |
| GSE29272 | GSM723523 | 60 | NA | 3 | NA | high-risk |
| GSE29272 | GSM723525 | 67 | NA | 3 | NA | high-risk |
| GSE29272 | GSM723527 | 59 | NA | 3 | NA | low-risk |
| GSE29272 | GSM723529 | 57 | NA | 2 | NA | high-risk |
| GSE29272 | GSM723531 | 51 | NA | 3 | NA | low-risk |
| GSE29272 | GSM723533 | 43 | NA | 3 | NA | high-risk |
| GSE29272 | GSM723535 | 55 | NA | 3 | NA | low-risk |
| GSE29272 | GSM723537 | 67 | NA | 2 | NA | high-risk |
| GSE29272 | GSM723539 | 44 | NA | 3 | NA | high-risk |
| GSE29272 | GSM723541 | 57 | NA | 3 | NA | high-risk |
| GSE29272 | GSM723543 | 47 | NA | 3 | NA | high-risk |
| GSE29272 | GSM723545 | 39 | NA | 3 | NA | low-risk |
| GSE29272 | GSM723547 | 63 | NA | 3 | NA | high-risk |
| GSE29272 | GSM723549 | 57 | NA | 3 | NA | high-risk |
| GSE29272 | GSM723551 | 49 | NA | 3 | NA | high-risk |
| GSE29272 | GSM723553 | 60 | NA | 3 | NA | low-risk |
| GSE29272 | GSM723555 | 59 | NA | 3 | NA | high-risk |
| GSE29272 | GSM723557 | 44 | NA | 1 | NA | high-risk |
| GSE29272 | GSM723559 | 68 | NA | 2 | NA | high-risk |
| GSE29272 | GSM723561 | 50 | NA | 3 | NA | high-risk |
| GSE29272 | GSM723563 | 63 | NA | 3 | NA | low-risk |
| GSE29272 | GSM723565 | 55 | NA | 3 | NA | high-risk |
| GSE29272 | GSM723567 | 62 | NA | 3 | NA | high-risk |
| GSE29272 | GSM723569 | 47 | NA | 3 | NA | high-risk |
| GSE29272 | GSM723571 | 43 | NA | 3 | NA | low-risk |
| GSE29272 | GSM723573 | 57 | NA | 2 | NA | low-risk |
| GSE29272 | GSM723575 | 66 | NA | 3 | NA | low-risk |
| GSE29272 | GSM723577 | 60 | NA | 3 | NA | low-risk |
| GSE29272 | GSM723579 | 64 | NA | 3 | NA | high-risk |
| GSE29272 | GSM723581 | 67 | NA | 1 | NA | low-risk |
| GSE29272 | GSM723583 | 33 | NA | 3 | NA | low-risk |
| GSE29272 | GSM723585 | 61 | NA | 3 | NA | low-risk |
| GSE29272 | GSM723587 | 60 | NA | 4 | NA | low-risk |
| GSE29272 | GSM723589 | 63 | NA | 3 | NA | low-risk |
| GSE29272 | GSM723591 | 61 | NA | 3 | NA | low-risk |
| GSE29272 | GSM723593 | 50 | NA | 1 | NA | high-risk |
| GSE29272 | GSM723595 | 54 | NA | 3 | NA | high-risk |
| GSE29272 | GSM723597 | 59 | NA | 1 | NA | low-risk |
| GSE29272 | GSM723599 | 54 | NA | 3 | NA | low-risk |
| GSE29272 | GSM723601 | 60 | NA | 3 | NA | low-risk |
| GSE29272 | GSM723603 | 42 | NA | 3 | NA | high-risk |
| GSE29272 | GSM723605 | 45 | NA | 3 | NA | low-risk |
| GSE29272 | GSM723607 | 68 | NA | 3 | NA | high-risk |
