# Supplementary material for: Integrated Analysis Identifies an Immune-Based Prognostic Signature for the Mesenchymal Identity in Gastric Cancer
Source: Biomed Res Int. 2020 Apr 9;2020:9780981. doi: 10.1155/2020/9780981 (PMC7171688; doi:10.1155/2020/9780981)
Supplement: Supplementary 12 — Table S4: GSEA results for the comparison of high- vs. low-risk groups. [file 9780981.f12.docx]

Table S4. GSEA results for the comparison of high- vs. low- risk groups

| **Pathway** | **P** | **ES** |
| --- | --- | --- |
| HALLMARK_TNFA_SIGNALING_VIA_NFKB | 0.0125 | 0.30 |
| HALLMARK_HYPOXIA | 0.0063 | 0.39 |
| HALLMARK_MITOTIC_SPINDLE | 0.0063 | 0.39 |
| HALLMARK_WNT_BETA_CATENIN_SIGNALING | 0.0174 | 0.48 |
| HALLMARK_TGF_BETA_SIGNALING | 0.0035 | 0.55 |
| HALLMARK_APOPTOSIS | 0.0053 | 0.40 |
| HALLMARK_NOTCH_SIGNALING | 0.0033 | 0.58 |
| HALLMARK_ESTROGEN_RESPONSE_LATE | 0.0048 | -0.37 |
| HALLMARK_MYOGENESIS | 0.0062 | 0.61 |
| HALLMARK_APICAL_JUNCTION | 0.0062 | 0.54 |
| HALLMARK_HEDGEHOG_SIGNALING | 0.0034 | 0.63 |
| HALLMARK_EPITHELIAL_MESENCHYMAL_TRANSITION | 0.0063 | 0.81 |
| HALLMARK_INFLAMMATORY_RESPONSE | 0.0063 | 0.34 |
| HALLMARK_XENOBIOTIC_METABOLISM | 0.0071 | -0.36 |
| HALLMARK_UV_RESPONSE_DN | 0.0048 | 0.62 |
| HALLMARK_ANGIOGENESIS | 0.0034 | 0.67 |
| HALLMARK_COAGULATION | 0.0048 | 0.36 |
| HALLMARK_IL2_STAT5_SIGNALING | 0.0063 | 0.40 |
| HALLMARK_KRAS_SIGNALING_UP | 0.0063 | 0.37 |
| HALLMARK_KRAS_SIGNALING_DN | 0.0095 | -0.35 |
| HALLMARK_PANCREAS_BETA_CELLS | 0.0014 | -0.63 |
| KEGG_GLYCOLYSIS_GLUCONEOGENESIS | 0.0067 | -0.48 |
| KEGG_FRUCTOSE_AND_MANNOSE_METABOLISM | 0.0112 | -0.52 |
| KEGG_STEROID_HORMONE_BIOSYNTHESIS | 0.0028 | -0.55 |
| KEGG_STARCH_AND_SUCROSE_METABOLISM | 0.0042 | -0.54 |
| KEGG_GLYCOSAMINOGLYCAN_BIOSYNTHESIS_CHONDROITIN_SULFATE | 0.0060 | 0.66 |
| KEGG_LINOLEIC_ACID_METABOLISM | 0.0015 | -0.67 |
| KEGG_GLYCOSPHINGOLIPID_BIOSYNTHESIS_LACTO_AND_NEOLACTO_SERIES | 0.0015 | -0.59 |
| KEGG_BUTANOATE_METABOLISM | 0.0085 | -0.52 |
| KEGG_RETINOL_METABOLISM | 0.0014 | -0.64 |
| KEGG_NITROGEN_METABOLISM | 0.0015 | -0.67 |
| KEGG_METABOLISM_OF_XENOBIOTICS_BY_CYTOCHROME_P450 | 0.0014 | -0.67 |
| KEGG_DRUG_METABOLISM_CYTOCHROME_P450 | 0.0014 | -0.59 |
| KEGG_DRUG_METABOLISM_OTHER_ENZYMES | 0.0056 | -0.53 |
| KEGG_MAPK_SIGNALING_PATHWAY | 0.0075 | 0.31 |
| KEGG_CALCIUM_SIGNALING_PATHWAY | 0.0062 | 0.36 |
| KEGG_MTOR_SIGNALING_PATHWAY | 0.0038 | 0.47 |
| KEGG_VASCULAR_SMOOTH_MUSCLE_CONTRACTION | 0.0051 | 0.56 |
| KEGG_WNT_SIGNALING_PATHWAY | 0.0056 | 0.37 |
| KEGG_TGF_BETA_SIGNALING_PATHWAY | 0.0044 | 0.52 |
| KEGG_AXON_GUIDANCE | 0.0051 | 0.42 |
| KEGG_FOCAL_ADHESION | 0.0067 | 0.62 |
| KEGG_ECM_RECEPTOR_INTERACTION | 0.0044 | 0.69 |
| KEGG_CELL_ADHESION_MOLECULES_CAMS | 0.0055 | 0.35 |
| KEGG_ADHERENS_JUNCTION | 0.0042 | 0.53 |
| KEGG_TIGHT_JUNCTION | 0.0054 | 0.34 |
| KEGG_GAP_JUNCTION | 0.0044 | 0.48 |
| KEGG_FC_GAMMA_R_MEDIATED_PHAGOCYTOSIS | 0.0044 | 0.40 |
| KEGG_LEUKOCYTE_TRANSENDOTHELIAL_MIGRATION | 0.0051 | 0.37 |
| KEGG_INTESTINAL_IMMUNE_NETWORK_FOR_IGA_PRODUCTION | 0.0042 | -0.53 |
| KEGG_OLFACTORY_TRANSDUCTION | 0.0012 | -0.52 |
| KEGG_TASTE_TRANSDUCTION | 0.0110 | -0.48 |
| KEGG_REGULATION_OF_ACTIN_CYTOSKELETON | 0.0067 | 0.46 |
| KEGG_PROGESTERONE_MEDIATED_OOCYTE_MATURATION | 0.0043 | 0.40 |
| KEGG_MATURITY_ONSET_DIABETES_OF_THE_YOUNG | 0.0015 | -0.68 |
| KEGG_LEISHMANIA_INFECTION | 0.0085 | 0.39 |
| KEGG_PATHWAYS_IN_CANCER | 0.0093 | 0.39 |
| KEGG_BASAL_CELL_CARCINOMA | 0.0039 | 0.44 |
| KEGG_MELANOMA | 0.0044 | 0.44 |
| KEGG_SMALL_CELL_LUNG_CANCER | 0.0044 | 0.44 |
| KEGG_HYPERTROPHIC_CARDIOMYOPATHY_HCM | 0.0044 | 0.61 |
| KEGG_ARRHYTHMOGENIC_RIGHT_VENTRICULAR_CARDIOMYOPATHY_ARVC | 0.0044 | 0.58 |
| KEGG_DILATED_CARDIOMYOPATHY | 0.0043 | 0.61 |
